# Multiconfigurational Ground State of a Diradicaloid Characterized at the Atomic Scale

**DOI:** 10.1021/jacs.5c13039

**Published:** 2025-10-17

**Authors:** Elia Turco, Lara Tejerina, Gonçalo Catarina, Andres Ortega-Guerrero, Nils Krane, Leo Gross, Michal Juríček, Shantanu Mishra

**Affiliations:** † nanotech@surfaces Laboratory, Empa—Swiss Federal Laboratories for Materials Science and Technology, Überlandstrasse 129, 8600 Dübendorf, Switzerland; ‡ Department of Chemistry, 27217 University of Zurich, Winterthurerstrasse 190, 8057 Zurich, Switzerland; § IBM Research Europe—Zurich, Säumerstrasse 4, 8803 Rüschlikon, Switzerland; ∥ Department of Physics, Chalmers University of Technology, 412 96 Gothenburg, Sweden

## Abstract

We report the tip-induced generation and scanning probe characterization of a singlet diradicaloid, consisting of two phenalenyl units connected by an sp-hybridized C_4_ chain on an ultrathin insulating NaCl surface. The bond-order contrast along the C_4_ chain measured by atomic force microscopy and mapping of charge-state transitions by scanning tunneling microscopy, in conjunction with multiconfigurational calculations, reveal that the molecule exhibits a many-body ground state. Our study experimentally demonstrates the manifestation of strong electronic correlations in the geometric and electronic structures of a single molecule.

## Graphical abstract

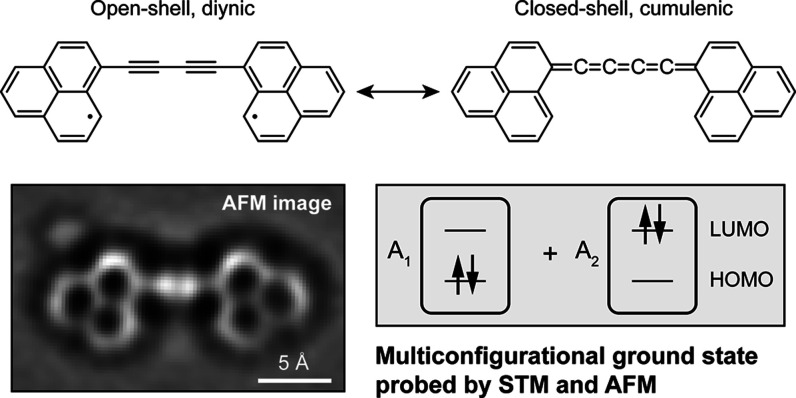

## Introduction

Diradicaloids represent an intriguing class of compounds whose reactivity and enigmatic electronic structure have fascinated chemists and physicists for over a century.^1–3^ As intermediates between diradicals and closed-shell molecules, their nature is central to understanding the chemical bond itself. Diradicaloids are represented as resonance hybrids between open- and closed-shell structures, which underscores the ambiguity in defining their electronic structure with theories that do not account for the multiconfigurational nature of electronic wave functions. The small gap between the highest occupied and lowest unoccupied molecular orbitals (HOMO and LUMO) of diradicaloids facilitates the mixing between multiple electronic configurations in the singlet ground state. This multiconfigurational nature is commonly quantified by the diradical index, which can take values between 0 (closed-shell) and 1 (diradical).^4,5^ A well-established correlation between aromatic stabilization and diradical character enables fine-tuning of the electronic and magnetic properties through structural design, making diradicaloids promising candidates for applications in optoelectronics and spintronics.^6^

Driven by progress in synthetic methods and improved characterization techniques, a number of singlet diradicaloids have been studied in recent years, for example, long acenes,^7–9^ periacenes,^10^ anthenes,^11–13^ zethrenes,^14–19^ rhombenes,^20,21^ and indenofluorenes.^22–25^ The advent of on-surface chemistry^26^ has extended the scope of the study of singlet diradicaloids to the atomic scale by means of scanning probe techniques. In this context, it is important to understand how the multiconfigurational ground state of singlet diradicaloids manifests in terms of the fundamental observables of a molecular system, such as bond order and molecular orbital densities.

In this study, we use atomic force microscopy (AFM) and scanning tunneling microscopy (STM) to generate and study a diradicaloid, **1** (C_30_H_16_, Figure 1), and elucidate the influence of electronic correlations on the geometric and electronic structures of the molecule. Compound **1** consists of two phenalenyl units (Figure 1a), sp^2^-conjugated polycyclic conjugated hydrocarbons with an *S* = 1/2 (doublet) ground state (*S* denotes the total spin quantum number), which are connected through their majority sublattice carbon atoms via an sp-hybridized C_4_ chain, resulting in an *S* = 0 (singlet) ground state as per Ovchinnikov’s rule.^27,28^

**Figure 1:**
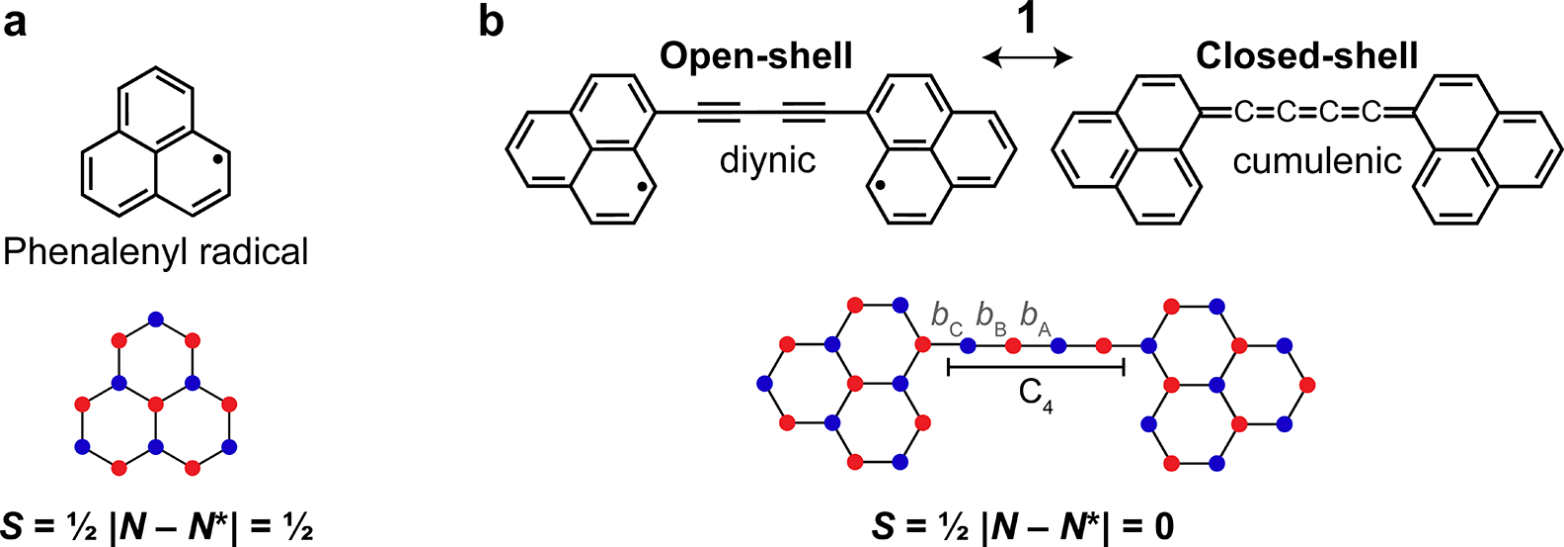
(a,b) Chemical structures and sublattice representations of phenalenyl radical (a) and compound **1** (b). The two sublattices are represented with different colors. *N* and N* denote the number of carbon atoms in the two sublattices. For **1**, two possible resonance structures are shown, namely, an open-shell with a diynic C_4_ chain, and a closed-shell with a cumulenic C_4_ chain. For the closed-shell resonance structure, the bond orders of *b*_A_, *b*_B_, and *b*_C_ should be similar, while for the open-shell resonance structure, *b*_B_ should have a higher bond order than *b*_A_ and *b*_C_.

As shown in Figure 1b, compound **1** can be represented as a resonance hybrid of two structures, namely, open- and closed-shell singlets. Importantly, the two resonance structures present different bonding motifs in the C_4_ chain, namely, diynic (alternating single and triple bonds) and cumulenic (all double bonds) for the open- and closed-shell structures, respectively. Previously, Hirao et al. studied a derivative of **1** in single-crystalline form.^29^ From X-ray structural analyses at temperatures between 100 and 250 K, the authors observed a bond-length alternation *b*_A_ − *b*_B_ > 0 at all temperatures (see Figure 1b for labeling), indicating contribution from the open-shell resonance form. The different bonding motifs for the two resonance structures make **1** a suitable system for characterization by AFM, which can distinguish C–C bonds of different bond orders.^30^ We show by AFM imaging that compared to a diynic bonding motif with formal C–C single and triple bonds, the C_4_ chain in **1** exhibits a markedly reduced bond-order contrast. Furthermore, STM imaging of **1** at the ion resonances reveals orbital densities that cannot be explained on the basis of charge-state transitions involving a single-determinant ground state of **1** (such as a closed-shell configuration with doubly occupied HOMO and empty LUMO). However, the experimental results can be explained well if one considers a ground state of **1** that is composed of multiple Slater determinants, that is, a multiconfigurational ground state.

## Results and Discussion

### Generation and Structural Characterization

Compound **1** was generated from the corresponding dihydro precursor **1p** (Figure 2a), which was synthesized in solution (Methods and Figures S1–S8). A submonolayer coverage of **1p** was sublimed on a Cu(111) surface partially covered by bilayer NaCl films (Figure S9). STM and AFM imaging showed the coexistence of both *E* and *Z* configurations of **1p**, which differ in the relative orientation of the two phenalene units through rotation about the C–C single bond connecting the phenalene units and the C_4_ chain. AFM imaging (Figures 2 and S10) revealed that both isomers adopt a mostly planar geometry on NaCl, and the sp-hybridized C_4_ chain exhibits a diynic bonding motif evidenced by a modulation of the frequency shift (Δ*f*) signal along the chain, as described below. Compound **1** was generated by applying voltage pulses to individual **1p** molecules by the tip of the STM/AFM system, which led to homolytic cleavage of the two C­(sp^3^)–H bonds.^31,32^ The sequential manipulation of **1p** to generate **1** was monitored by AFM imaging, with an example shown in Figure S11. In the main text, we focus on the characterization of (*Z*)-**1**. The characterization of (*E*)-**1**, which is electronically and structurally (with respect to the C_4_ chain) equivalent to (*Z*)-**1**, is presented in the Supporting Information.

**Figure 2:**
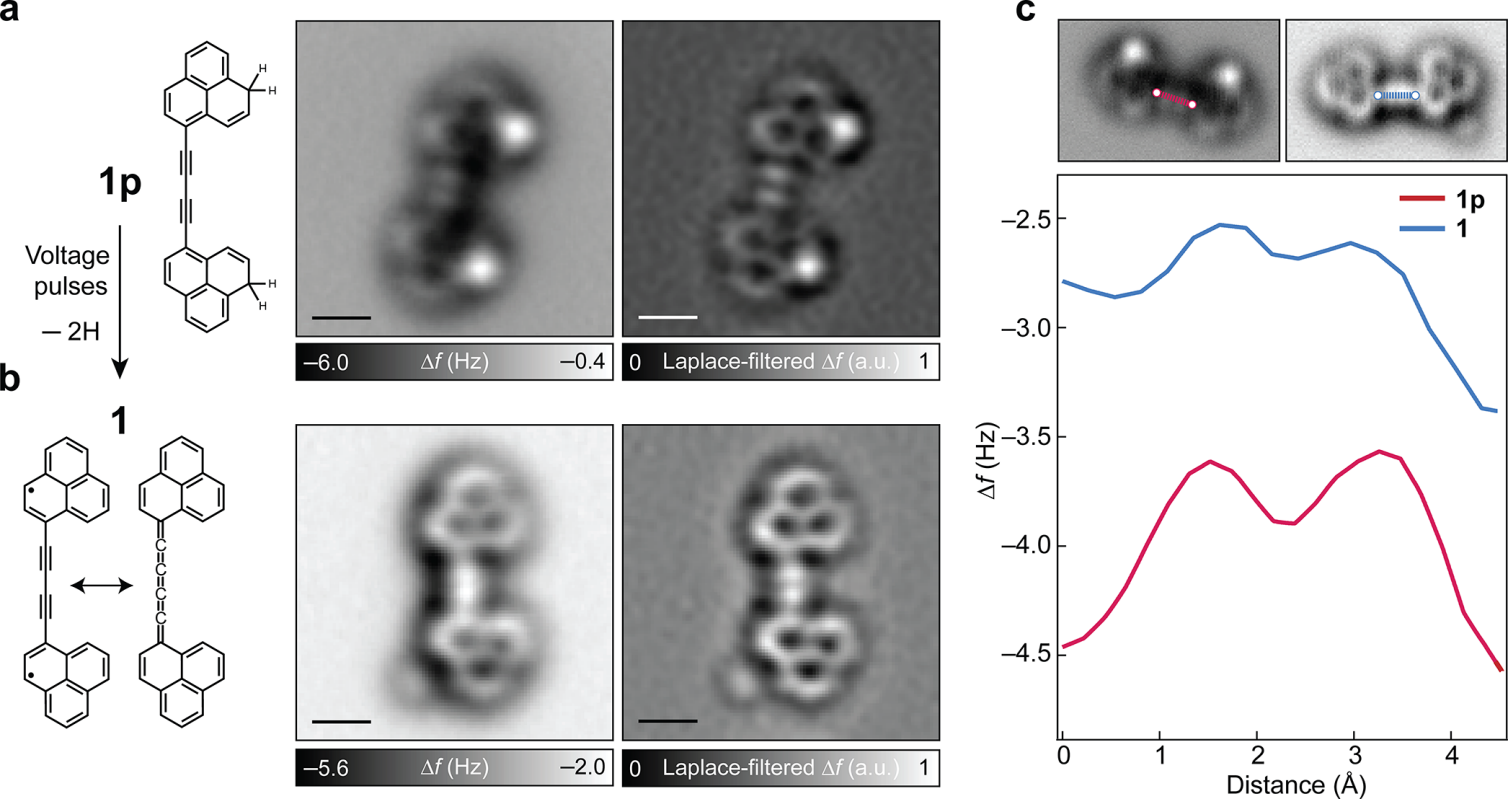
Structural characterization of **1p** and **1**. (a,b) From left to right: chemical structures, AFM images, and corresponding Laplace-filtered AFM images of **1p** (a) and **1** (b); STM set-point: *V* = 0.2 V and *I* = 1.0 pA on NaCl, tip height Δ*z* = 0.5 Å. a.u. denotes arbitrary units. (c) Δ*f* line profiles along the C_4_ chains of **1p** (red) and **1** (blue). Scale bars: 0.5 nm.

We now focus on elucidating the difference in the bond-order contrasts of the C_4_ chain in **1p** and **1**. In AFM imaging, chemical bonds with higher bond orders show a larger Δ*f* signal due to stronger repulsive forces. Figure 2a shows an AFM image of **1p**, where two bright features that correspond to the formal C–C triple bonds^33,34^ labeled *b*_B_ in Figure 1b (that exhibit a higher bond order than the neighboring formal C–C single bonds labeled *b*_A_ and *b*_C_ in Figure 1b) are evident at the center of the C_4_ chain. Note that the two bright features at the phenalene units correspond to the dihydro groups. Compared to **1p**, there is a noticeable decrease in the bond-order contrast of the C_4_ chain in AFM imaging of **1** (Figure 2b, see also Figures S11 and S12). This difference in the bond-order contrast is also visualized in the Δ*f* line profiles along the C_4_ chains of **1p** and **1** (Figure 2c), where **1p** exhibits a larger Δ*f* modulation compared to **1**. However, the fact that a Δ*f* modulation remains in **1** indicates that the C_4_ chain in **1** is neither diynic with formal C–C single and triple bonds (as in **1p**) nor cumulenic (where no bond order contrast should be visible^35^). We note that changes of the bond order of a molecule have previously been observed due to chemisorption on a metallic surface.^36^ However, such effects due to interaction with the surface are not expected in our work because of the adsorption of **1** on the inert NaCl surface, where the molecule is physisorbed.

### Electronic Characterization

Based on AFM imaging of **1** that reveals bond-order contrast in the C_4_ chain that is intermediate between diynic (corresponding to a diradical state with two singly occupied molecular orbitals) and cumulenic (corresponding to a closed-shell state with doubly occupied HOMO and empty LUMO) geometries, and the small DFT-calculated HOMO–LUMO gap of **1** (Figure S13), it is likely that the system exhibits a multiconfigurational ground state. In line with this expectation, STM imaging of **1** at voltages corresponding to the ion resonances reveals orbital densities that cannot be explained with a single-reference picture but requires invoking a multiconfigurational framework, as discussed below. Here, tunneling events at the ion resonances are considered as many-body transitions between different charge states of **1**, and the electronic ground state of **1** is described by a weighted combination of multiple Slater determinants.

Figure 3a (see also Figures S14–S17) shows a differential conductance spectrum (d*I*/d*V*(*V*), where *I* and *V* denote the tunneling current and bias voltage, respectively) acquired on **1**, exhibiting three peaks centered at −1.8, 0.9, and 1.5 V, labeled positive ion resonance (PIR), negative ion resonance (NIR), and NIR+1, respectively. In a single-reference picture and assuming a closed-shell electronic configuration, the peak at −1.8 V should correspond to electron detachment from the HOMO of **1**. As shown in Figure S18, the calculated HOMO local density of states (LDOS) map exhibits a maximum at the center of the C_4_ chain. Although the STM image at −1.8 V (Figure 3c) shows a high intensity at the center of the C_4_ chain, there is a concomitant depression reminiscent of a nodal plane, which is not explained by a transition involving only the HOMO. At positive biases, the resonance at 0.9 V should correspond to electron attachment to the LUMO of **1**, and in this case, the LUMO LDOS map (Figure S18) agrees well with the STM image at 0.9 V. However, for the resonance at 1.5 V, which should correspond to electron attachment to the LUMO+1 of the molecule, the corresponding STM image should show the superposition of LUMO and LUMO+1 densities (because electron attachment to both LUMO and LUMO+1 is possible at this bias). The STM image at 1.5 V does not agree well with the LDOS map corresponding to the superposition of the LUMO and LUMO+1 (Figure S18), but counterintuitively, agrees better with the LDOS map corresponding to the superposition of the HOMO and LUMO. Clearly, a single-reference picture fails to account for these peculiar features in the STM images.

**Figure 3:**
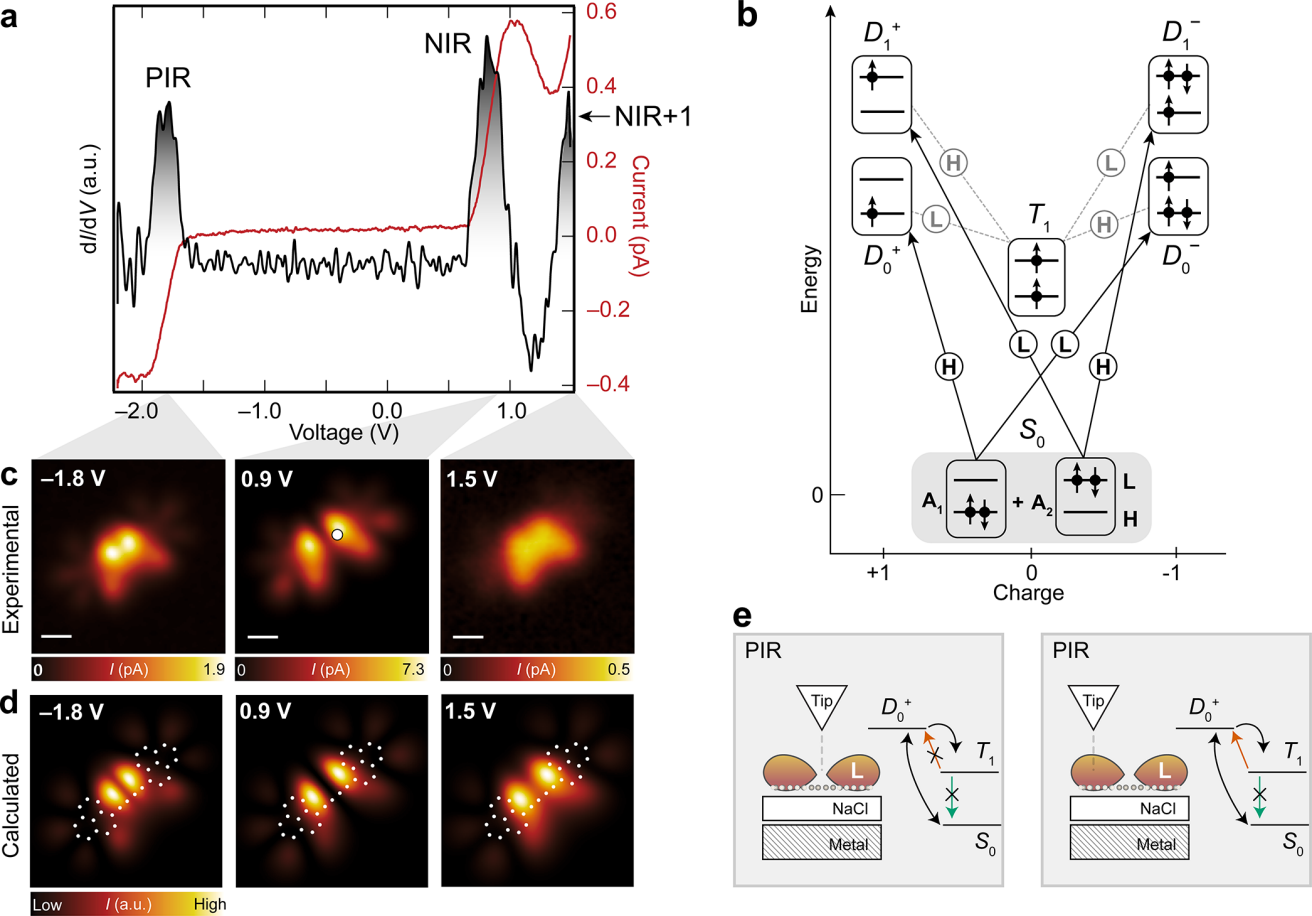
Electronic characterization of **1**. (a) Constant-height *I*(*V*) spectrum and the corresponding d*I*/d*V*(*V*) spectrum acquired on **1** (open feedback parameters on the molecule: *V* = −2.2 V, *I* = 0.4 pA). The acquisition position is indicated by the filled white circle in (c). PIR and NIR denote the positive and negative ion resonances, respectively. (b) Scheme of the many-body transitions relevant for the measured ion resonances. The transitions are labeled according to the orbital involved: H (HOMO) or L (LUMO). The *S*_0_ → *D*_1_^+^ transition was not accessible in the experimental voltage range. (c) Constant-height STM images at the ion resonances (from left to right, open feedback parameters on NaCl: *V* = −1.8 V, *I* = 1.0 pA, Δ*z* = 1.9 Å; *V* = 0.9 V, *I* = 1.0 pA, Δ*z* = 2.3 Å; *V* = 1.5 V, *I* = 1.0 pA, Δ*z* = 3.5 Å). (d) Transition probability maps at the voltages corresponding to the experimental images in (c) (see Figures S18, S23, and S24). The molecular structure of **1** is overlaid on the maps. (e) Sketch illustrating triplet trapping for tip positions above the LUMO nodal plane (left), and no trapping for tip positions above finite LUMO density (right). The data in (a,c) were acquired with a metallic tip. Scale bars: 0.5 nm.

The experimental STM images can be reconciled with measured orbital densities if one instead considers a multiconfigurational picture, as demonstrated previously for a similar case.^37^ Within a minimal multiconfigurational framework, the neutral singlet ground state of **1** (*S*_0_) is described as a linear combination of two Slater determinants ψ_B_ and ψ_AB_

1 $$S_{0}=A_{1}\psi_{B}+A_{2}\psi_{AB}$$

where ψ_B_ = |↑↓⟩_HOMO_|0⟩_LUMO_ and ψ_AB_ = |0⟩_HOMO_|↑↓⟩_LUMO_ correspond to electronic configurations with bonding (doubly occupied HOMO and empty LUMO) and antibonding (empty HOMO and doubly occupied LUMO) symmetries, respectively. To validate our assumption about the multiconfigurational ground state of **1** (eq 1), we performed calculations using the density matrix renormalization group (DMRG) for the Hubbard model as well as complete active space self-consistent field (Methods, Figures S13 and S19–S23, and Tables S1–S4). All calculations were done for three distinct geometries of **1**: a DFT PBE0-XC UKS optimized structure, a cumulenic structure with all double bonds (from PBE0 optimized ethylene) in the C_4_ chain, and a diynic structure with alternating single and triple bonds (from PBE0 optimized propyne) in the C_4_ chain (Tables S5–S7). For brevity, we will focus on the DMRG results, yielding the most accurate description^38^ of the multiconfigurational nature of **1**. The calculations corroborate the assumption of a singlet ground state with a predominantly bonding character (|*A*_1_|^2^/|*A*_2_|^2^ > 4) across all considered geometries. The weight of the doubly excited configuration, |*A*_2_|^2^, ranges from 0.04 to 0.12, increasing from the cumulenic to the diynic structure. For the DFT PBE0-XC UKS optimized structure, which is the focus of the following analysis, we obtain |*A*_1_|^2^ = 0.60 and |*A*_2_|^2^ = 0.06. Since |*A*_1_|^2^ + |*A*_2_|^2^ < 1, the ground state *S*_0_ involves more Slater determinants than suggested by the simplified picture of eq 1. However, as we show, this two-configuration picture describes well the observed electronic properties of **1**.

Figure 3b illustrates a schematic of the many-body electronic transitions corresponding to the measured ion resonances of **1**. To elucidate the excitation mechanisms responsible for the experimental STM images in Figure 3c, we modeled the system using a master equation (Methods and Figure S24 and Table S4) that incorporates all relevant tunneling pathways and a finite lifetime of the neutral excited states. The spatially resolved transition probability maps (Figure 3d) associated with the transitions depicted in Figure 3b were derived from Dyson orbitals computed via DMRG (Figures S18 and S22–S24). For this, we assumed tunneling with an s-wave tip because of the predominant s-wave tunneling character at large tip–sample distances, even for carbon monoxide-functionalized tips.^39^ Starting from *S*_0_, we assign the positive ion resonance at −1.8 V to a resonant transition to the cationic doublet ground state *D*_0_^+^, wherein an electron is detached from the HOMO. The system subsequently decays to *S*_0_ by electron transfer from the surface, resulting in a net current. However, the corresponding *S*_0_ → *D*_0_^+^ Dyson orbital (Figures S22 and S23) does not feature a central nodal plane, as observed in the STM image at −1.8 V. To resolve this discrepancy, one must consider the role of the neutral triplet excited state *T*_1_. Following an initial *S*_0_ → *D*_0_^+^ resonant tunneling event, the system can be neutralized via electron transfer from the surface in two different ways. By electron attachment to the HOMO, the system decays to *S*_0_, while by electron attachment to the LUMO, the system decays to *T*_1_ (located ∼0.36 eV above *S*_0_ according to DMRG calculations). If the system is in *T*_1_, it can either decay to *S*_0_ (which is a slow process because it requires a change in the spin multiplicity), or the system can be excited to *D*_0_^+^ via electron tunneling to the tip, followed by a decay to *S*_0_. As shown schematically in Figure 3e, the amplitude of the *T*_1_ → *D*_0_^+^ transition, which involves electron detachment from the LUMO, is strongly dependent on the tip position. If the tip is located at the center of the C_4_ chain (where the LUMO exhibits a nodal plane), the system is trapped in *T*_1_. The tunneling channel through the molecule is effectively blocked, and the total current is reduced at the nodal plane, as observed in the STM image. Away from the chain center, where the LUMO has a nonzero amplitude, this transition can take place. The corresponding transition probability map, which takes into account the *S*_0_ → *D*_0_^+^ and *T*_1_ → *D*_0_^+^ transitions (Figure 3d, see also Figure S23 for the relative intensities of the transitions), exhibits good agreement with the STM image at −1.8 V. We note that transitions involving ground and excited states of a molecule have been previously predicted for copper phthalocyanine,^40^ and experimentally observed for several molecular systems by STM/AFM-based spectroscopy^37,41–43^ and STM-induced luminescence measurements.^44,45^

The NIR at 0.9 V represents a resonant transition from *S*_0_ to the anionic doublet ground state *D*_0_^–^, corresponding to electron attachment to the LUMO. The Dyson orbital for this transition features the characteristic central nodal plane as seen in the experiment. The system may subsequently decay to either *S*_0_ or *T*_1_, as described above. The decay to *T*_1_ opens an additional tunneling channel via the *T*_1_ → *D*_0_^–^ transition, which does not feature a central nodal plane (as it involves electron attachment to the HOMO). However, this pathway does not alter the appearance of the STM image because if the tip is positioned above the nodal plane of the *S*_0_ → *D*_0_^–^ transition, the system is not excited to *D*_0_^–^ and, therefore, cannot decay to *T*_1_. The transition probability map that takes into account the *S*_0_ → *D*_0_^–^ and *T*_1_ → *D*_0_^–^ transitions reproduces the experimental STM image at 0.9 V. The NIR+1 at 1.5 V corresponds to a resonant transition from *S*_0_ to the anionic doublet excited state *D*_1_^–^, where an electron is attached to the HOMO. This process becomes possible because of the multiconfigurational ground state of **1**, where the ψ_AB_ component of *S*_0_ contributes. The transition probability map, which, besides the resonant *S*_0_ → *D*_1_^–^ transition, includes contributions from the off-resonant *S*_0_ → *D*_0_^–^ transition (accessible at 1.5 V but with reduced spectral weight), and the *T*_1_ → *D*_0_^–^ and *T*_1_ → *D*_1_^–^ transitions, exhibits good agreement with the experimental STM image at 1.5 V.

The explanations for the effects of the multiconfigurational ground state on STM orbital density images were brought forward by Yu et al.^37^ Here, in addition to the STM orbital density images, we also observe signatures of the multiconfigurational ground state of a molecule by AFM, revealing contributions from both cumulenic and diynic resonance structures.

## Conclusions

We presented the generation and characterization of a neutral diradicaloid **1**, revealing experimental signatures and observables related to its multiconfigurational ground state in scanning probe measurements. We showed that the maps of charge-state transitions of **1** measured by STM cannot be explained by a picture wherein electron detachment or attachment takes place in the framework of single-particle states, but can only be explained if the ground state of **1** is considered to be a multiconfigurational state consisting of a weighted combination of multiple Slater determinants. Moreover, and in line with the observations of the STM measurements, AFM imaging reveals that the C_4_ bridge of **1** exhibits a bond-order contrast that is intermediate between diynic and cumulenic bonding motifs, lending support to the picture that **1** is neither a diradical nor a closed-shell system but a diradicaloid best described as a resonance hybrid of open- and closed-shell states. Our study thus provides a striking example of strong electronic correlations manifesting at the atomic scale in real space.

## Supplementary Material



## Data Availability

The raw NMR data are available free of charge on the public repository Zenodo under the link: https://zenodo.org/record/15311165 (DOI: 10.5281/zenodo.15311165).

## Notes

Experimental and theoretical methods, additional scanning probe and theoretical data, atomic coordinates of the three geometries of **1**, and solution synthesis and characterization (PDF)

## ⊥

QuTech and Kavli Institute of Nanoscience, Delft University of Technology, 2600 GA Delft, The Netherlands
