# Supplementary material for: Multiconfigurational Ground State of a Diradicaloid Characterized at the Atomic Scale
Source: J Am Chem Soc. 2025 Oct 17;147(43):39616–22. doi: 10.1021/jacs.5c13039 (PMC12576812; doi:10.1021/jacs.5c13039)
Supplement: Supplementary file 1 [file ja5c13039_si_001.pdf]

Supporting Information: The Multiconfigurational Ground State of a Diradicaloid Characterized at the Atomic Scale

Elia Turco,[*,†,‖] Lara Tejerina,[‡,⊥] Gonçalo Catarina,[†,⊥] Andres Ortega-Guerrero,[†] Nils Krane,[†] Leo Gross,[¶] Michal Juríček,[‡] and Shantanu Mishra[*,¶,§]

†*nanotech@surfaces Laboratory, Empa – Swiss Federal Laboratories for Materials Science and Technology, Überlandstrasse 129, 8600 Dübendorf, Switzerland*

‡*Department of Chemistry, University of Zurich, Winterthurerstrasse 190, 8057 Zurich, Switzerland*

¶*IBM Research Europe – Zurich, Säumerstrasse 4, 8803 Rüschlikon, Switzerland*

§*Department of Physics, Chalmers University of Technology, 412 96 Gothenburg, Sweden*

‖*Current address: QuTech and Kavli Institute of Nanoscience, Delft University of Technology, 2600 GA Delft, The Netherlands*

⊥*These authors contributed equally to this work.*

E-mail: eturco@tudelft.nl; shantanu.mishra@chalmers.se

I Methods: solution synthesis and characterization

General information. Glassware utilized in the reactions, carried out under both anhydrous and non-anhydrous conditions, were cleaned and dried in an oven at 150 °C for at least 24 h prior to the experiment. All reagents and solvents, including non-anhydrous and anhydrous solvents such as CH_2Cl_2, cyclohexane, EtOAc or THF, were supplied from commercial sources and used without additional purification unless otherwise noted. Thin-layer chromatography (TLC) was used to monitor the reactions, using aluminium sheets covered with silica gel containing fluorescent indicator UV254 (available from Alugram SIL G/UV254, Macherey-Nagel or Sigma-Aldrich) and viewed under UV light (254 or 365 nm). Silica gel 60 (230–400 mesh, Sigma-Aldrich) was used for flash column chromatography.

All ^{1}H and ^{13}C NMR spectra were recorded at 25 °C on a Bruker 400 MHz spectrometer. Chemical shifts (δ) are reported in parts per million (ppm) relative to the solvent residual peak: $CDCl_3$ ($\delta = 7.26$ ppm for ^{1}H and 77.16 ppm for ^{13}C).[1]

Electrospray ionisation (ESI) high-resolution mass spectra (HRMS) were recorded on a timsTOF Pro TIMS-QTOF-MS instrument (Bruker Daltonics GmbH, Bremen, Germany). The samples were dissolved (e.g., in MeOH) at a concentration of approximately 50 μg mL^{-1} and analyzed via continuous flow injection (2 μL min^{-1}). The mass spectrometer was operated in the positive (or negative) electrospray ionization mode at 4,000 V (–4,000 V) capillary voltage and –500 V (500 V) endplate offset with a nitrogen nebulizer pressure of 0.4 bar and a dry gas flow of 4 L min^{-1} at 180 °C. Mass spectra were acquired in a mass range from m/z 50 to 2,000 at approximately 20,000 resolution (m/z 622) and at 1.0 Hz rate. The mass analyzer was calibrated between m/z 118 and 2,721 using an Agilent ESI-L low concentration tuning mix solution (Agilent, USA) at a resolution of 20,000, giving a mass accuracy below 2 ppm. All solvents used were purchased in best LC-MS quality.

The reaction was carried out under inert conditions. To a cooled (–78 °C) solution of (triisopropylsilyl)acetylene (TIPSA; 0.99 mL, 4.4 mmol) in THF (5 mL), *n*-BuLi (2.2 mL,

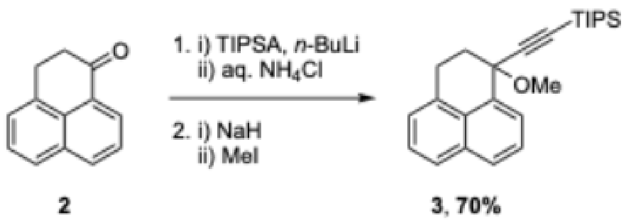

Figure S1:
Synthesis of triisopropyl((1-methoxy-2,3-dihydro-1H-phenalen-1-yl)ethynyl)silane (**3**).

3.5 mmol, 1.6 M in hexanes) was added dropwise over 10 min and the reaction mixture was stirred at –78 °C for 2 h. Then, a solution of 2,3-dihydro-1H-phenalen-1-one (2; 0.20 g, 1.1 mmol) in THF (6 mL) was added dropwise at –78 °C, and the mixture was stirred for 15 min before it was allowed to warm up to room temperature and stirred for additional 2 h. The reaction mixture was poured in ice-cold aq. NH_4Cl (sat.) and extracted with diethyl ether (3 × 40 mL). The combined organic layers were dried over anhydrous $MgSO_4$ and filtered, and the solvent was evaporated under vacuum. The residue was passed through a silica plug (CH_2Cl_2) to yield a hydroxy intermediate as a colorless oil that was used in step 2 without further purification.

To a solution of the hydroxy intermediate dissolved in THF (9 mL), NaH (0.28 g, 6.9 mmol, 60% dispersion in mineral oil) was added. The reaction mixture was stirred for 30 min at room temperature before methyl iodide (0.61 g, 0.27 mL, 4.3 mmol) was added and the reaction mixture was stirred at 40 °C for 16 h. Then, the mixture was filtered through a celite plug and the solvent was evaporated under vacuum. The residue was purified by column chromatography (SiO_2, cyclohexane to cyclohexane/ethyl acetate 200:1) to afford the desired product as a yellow-brown oil (293 mg, 0.774 mmol) in 70% yield over the two steps.

^{1}**H NMR** (400 MHz, $CDCl_3$, ppm): δ 7.89 (dd, J = 7.1, 1.2 Hz, 1H), 7.76 (dd, J = 8.3, 1.2 Hz, 1H), 7.63 (d, J = 8.2 Hz, 1H), 7.41 (dd, J = 8.3, 7.1 Hz, 1H), 7.33 (dd, J = 8.2, 7.0 Hz, 1H), 7.23 (d, J = 7.0 Hz, 1H), 3.40 (ddd, J = 16.9, 12.8, 4.7 Hz, 1H), 3.27 (s, 3H), 2.95 (ddd, J = 16.3, 4.0, 3.9 Hz, 1H), 2.57 (ddd, J = 13.4, 4.7, 3.3 Hz, 1H), 2.30 (ddd, J = 13.1,

13.1, 4.6 Hz, 1H), 1.08–1.03 (m, 21H).

^{13}**C NMR** (101 MHz, $CDCl_3$): δ 134.8, 134.0, 133.3, 129.1, 128.2, 126.2, 125.7, 125.6, 124.93, 124.91, 107.7, 88.1, 74.3, 51.4, 35.5, 25.5, 18.8, 11.4.

HRMS (ESI): m/z: $[M + Na]^+$ Calcd for $C_{25}H_{34}OSi$ 401.2271; Found 401.2265, $[M + K]^+$ Calcd for $C_{25}H_{34}OSi$ 417.2011; Found 417.2005.

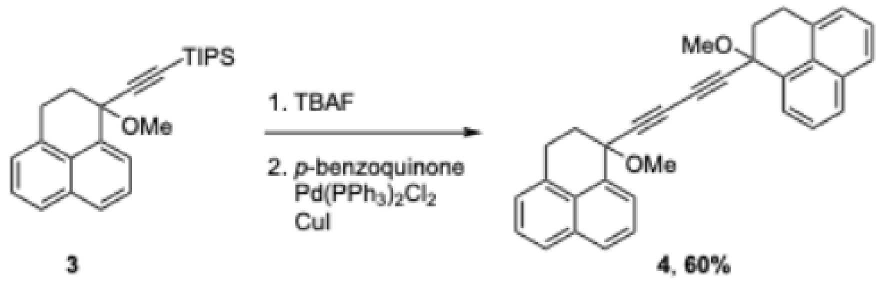

Figure S2: 1,4-Bis(1-methoxy-2,3-dihydro-1H-phenalen-1-yl)buta-1,3-diyne (**4**).

The reaction was carried out under inert conditions. To a solution of compound **3** (293 mg, 0.774 mmol) in dichloromethane (30 mL), TBAF (4.64 mL, 4.64 mmol, 1 M solution in THF) was added at room temperature and the reaction mixture was stirred for 30 min before it was filtered through a silica plug (CH_2Cl_2) and the solvent was evaporated under vacuum. The alkyne intermediate was used immediately in step 2 without further purification.

To a solution of the alkyne intermediate in toluene (50 mL), diisopropylamine (1 mL) was added and the mixture was stirred for 15 min. Then, $Pd(PPh_3)_2Cl_2$ (5.44 mg, 7.75 μmol), CuI (14.8 mg, 77.5 μmol), and *p*-benzoquinone (33 mg, 0.31 mmol) were added, and the reaction mixture was stirred at 50 °C for 2 h. The solvent was evaporated under vacuum and the residue was purified by column chromatography (SiO_2, cyclohexane to cyclohexane/ethyl acetate 100:1) to afford the desired product as a red solid (101 mg, 0.228 mmol) in 60% yield over the two steps.

^{1}**H NMR** (400 MHz, $CDCl_3$, ppm): δ 7.90 (dd, J = 7.2, 1.2 Hz, 2H), 7.86 (dd, J = 8.3, 1.3 Hz, 2H), 7.73 (d, J = 8.2 Hz, 2H), 7.51 (dd, J = 8.3, 7.1 Hz, 2H), 7.43 (dd, J = 8.2, 7.0 Hz, 2H), 7.33 (dd, J = 6.9, 1.3 Hz, 2H), 3.44 (ddd, J = 16.3, 11.5, 4.6 Hz, 2H), 3.37 (s, 6H), 3.10 (ddd, J = 16.4, 4.6, 4.6 Hz, 2H), 2.59 (ddd, J = 13.1, 4.6, 4.6 Hz, 2H), 2.41 (ddd, J =

13.1, 11.6, 4.6 Hz, 2H).

^{13}C NMR (101 MHz, $CDCl_3$): δ 134.4, 134.0, 132.8, 129.4, 128.0, 126.3, 125.8, 125.6, 125.1, 125.0, 80.2, 74.6, 71.2, 51.9, 34.8, 25.7.

HRMS (ESI): m/z: $[M + Na]^+$ Calcd for $C_{32}H_{26}O_2$ 465.1825; Found 465.18304.

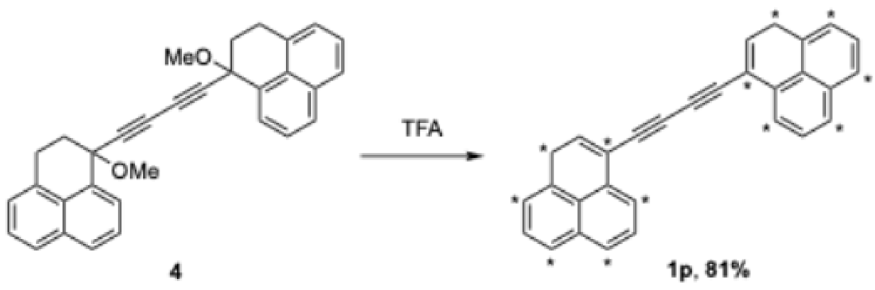

Figure S3: 1,4-Di(1H-phenalen-3-yl)buta-1,3-diyne (**1p**).

The reaction was performed under inert conditions. Compound **4** (16 mg, 36 μmol) was dissolved in argon-saturated $CDCl_3$ (0.6 mL) in an NMR tube. Trifluoroacetic acid (TFA; 4.0 μL, 54 μmol) was then added, and the tube was gently shaken at room temperature, leading to an immediate darkening of the solution. The reaction progress was monitored by ^{1}H NMR spectroscopy. The signals corresponding to compound **4** disappeared within 25 min; however, the reaction was monitored for a total of 2 h. The solvents, including methanol (formed as a side product), were evaporated directly from the NMR tube to afford the desired product (11 mg, 29 μmol, 81%) as a solid. The product was stored under argon in the original NMR tube and kept in the freezer. The sample was only freshly opened prior to surface studies. Several attempts to purify the compound via short-column chromatography (SiO_2 or Al_2O_3) under inert conditions were unsuccessful, as the product decomposed during the process. Due to its high sensitivity, high-resolution mass spectrometry (HRMS) could not be successfully performed.

^{1}H NMR (400 MHz, $CDCl_3$): Compound **1p** is obtained as a mixture of regioisomers, which differ by positions of the methylene groups that can occupy any α-position (marked with asterisks) of the phenalenyl subunit. This mixture of regioisomers gives a complex ^{1}H NMR spectrum with characteristic signals for the methylene groups ($\sim$4 ppm). The ratio

between the integrated signal intensity of all methylene groups and all aromatic protons is ∼2:7, matching the expected value.

Copies of NMR spectra:

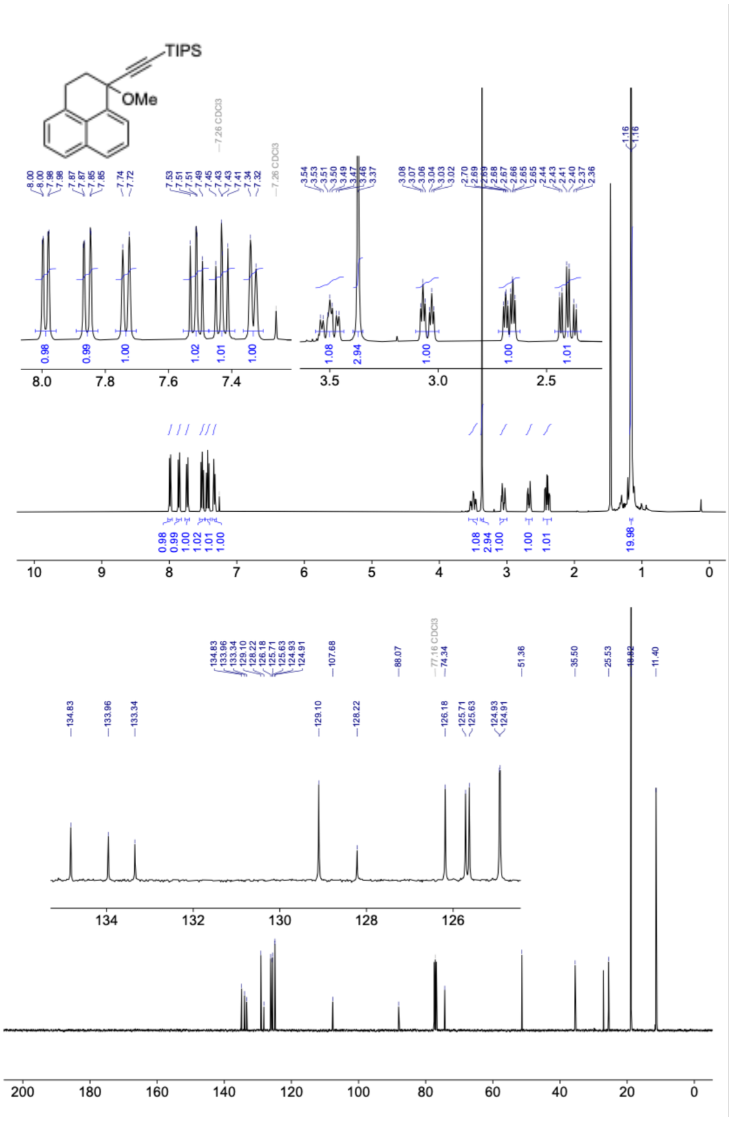

Figure S4: ^{1}H (top) and ^{13}C NMR (bottom) copies for compound **3**, $CDCl_3$, 400 MHz.

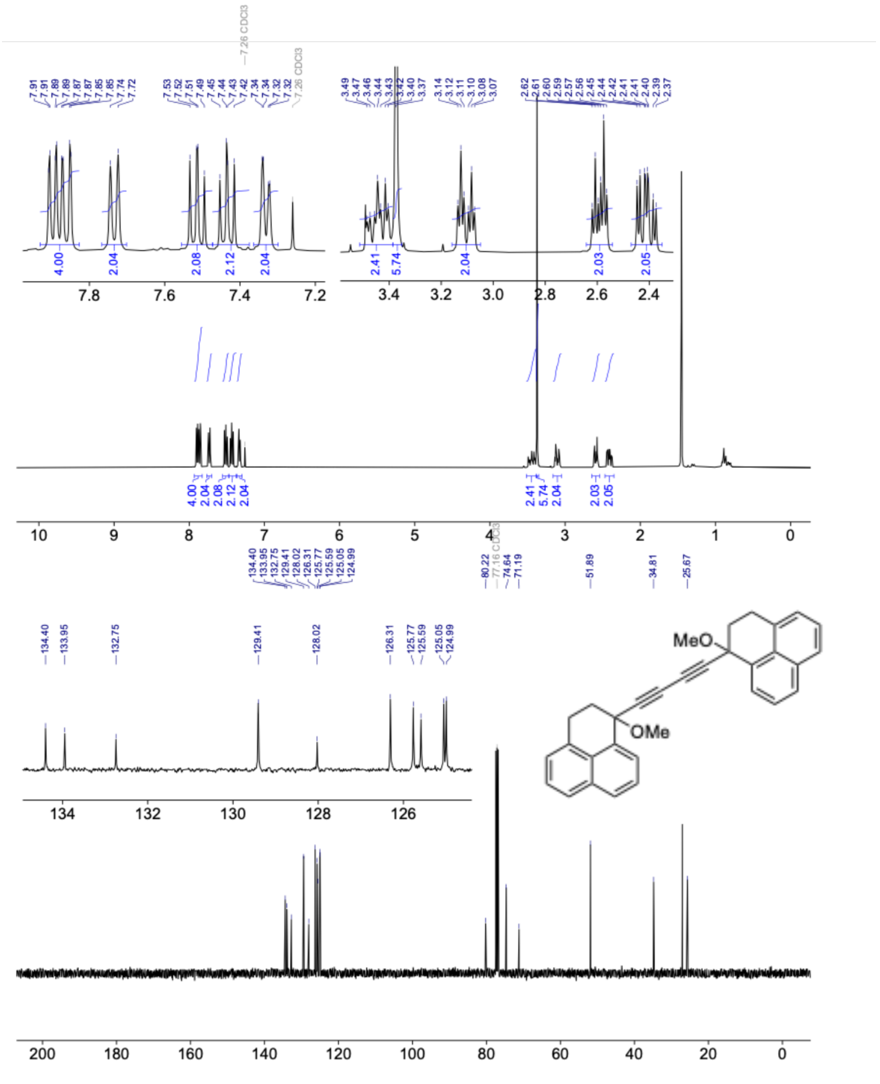

Figure S5: ^{1}H (top) and ^{13}C NMR (bottom) copies for compound **4**, $CDCl_3$, 400 MHz.

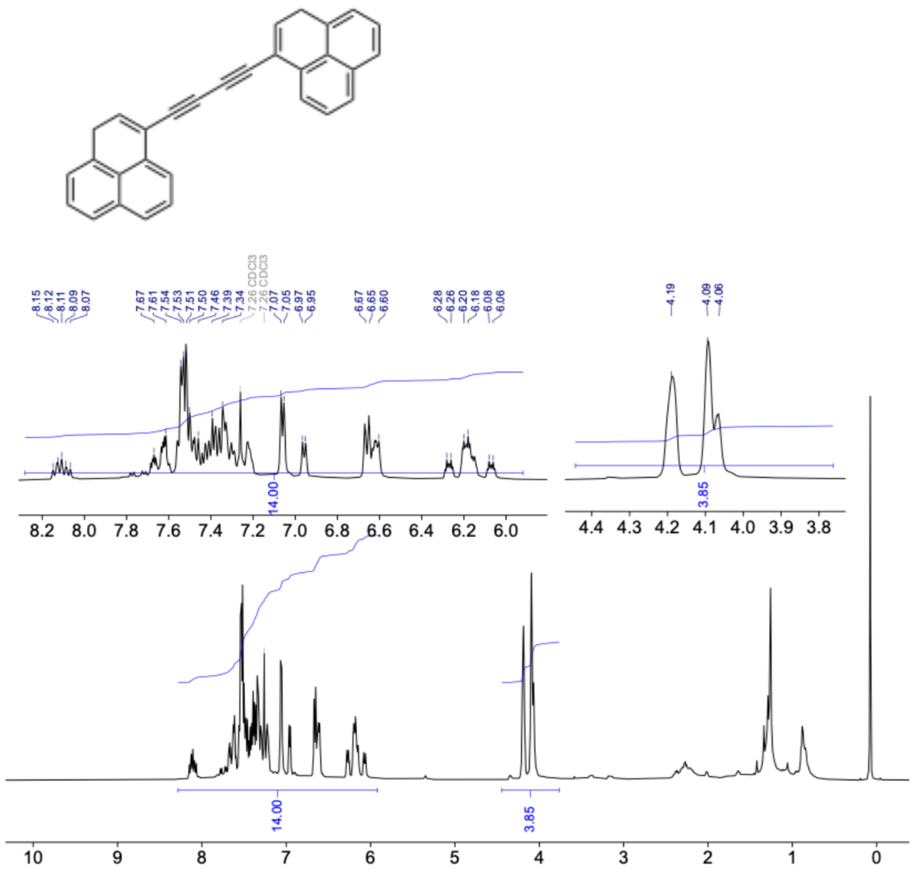

Figure S6: ^{1}H NMR copy for compound **1p**, $CDCl_3$, 400 MHz.

Copies of mass spectra:

HR-ESI Report

Analysis Info

Analysis Name	D:\Data\UZH_Data\Service\Data\20_juhres_0123.d	Acquisition Date	7/9/2020 7:32:58 AM
Method	Service_Syringe_Pump_Low_Mass_Range_pos.m	Operator	Demo User
Sample Name	LT224B	Instrument	timsTOF Pro 1854399.00195
Comment	Client: Tejerina<br>Solvent: MeOH + NaI		

Acquisition Parameter

Source Type	ESI	Ion Polarity	Positive	Set Nebulizer	0.3 Bar
Focus	Active			Set Dry Heater	180 °C
Scan Begin	50 m/z	Set Capillary	4500 V	Set Dry Gas	4.0 l/min
Scan End	2000 m/z	Set End Plate Offset	-500 V	Set Divert Valve	Source

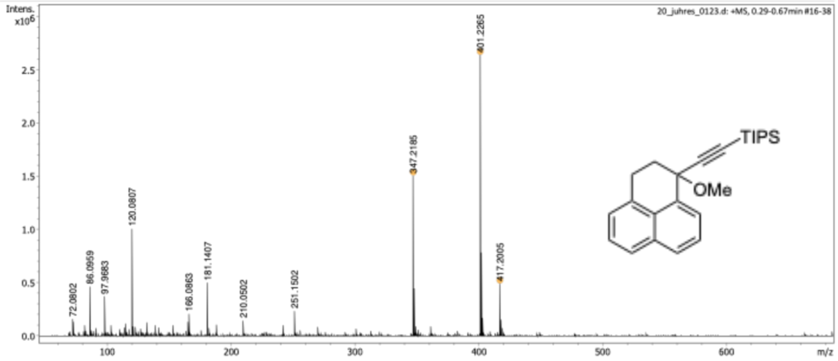

HR-ESI Report

Meas. m/z	Ion Formula	Sum Formula	m/z	err [ppm]	|err| [mDa]	mSigma	# mSigma	Score	rdb	e⁻ Conf	N-Rule
347.2185	C24H31Si	C24H31Si	347.2190	1.3	0.5	12.7	1	100.00	11.0	even	ok
401.2265	C22H33N2O3Si	C22H33N2O3Si	401.2255	-2.5	1.0	9.0	1	96.86	9.0	even	ok
	C25H34NaOSi	C25H34NaOSi	401.2271	1.5	0.6	17.6	2	100.00	10.0	even	ok
	C31H29	C31H29	401.2264	-0.3	0.1	24.5	3	81.51	18.0	even	ok
417.2005	C25H34KOSi	C25H34KOSi	417.2011	1.4	0.6	3.0	1	100.00	10.0	even	ok
	C25H29N2O2Si	C25H29N2O2Si	417.1993	-2.9	1.2	35.6	2	28.36	14.0	even	ok

Initial calibration from acquisition

Date:	7/9/2020 7:21:58 AM
Polarity:	Positive
Reference mass list:	Tuning Mix ES-TOF (ESI)
Calibration mode:	HPC Calibration
Standard deviation:	n.a.

Reference m/z	Resulting m/z	Intensity	Error [ppm]
118.0863	118.0863	405013	-0.000
322.0481	322.0481	153601	-0.000
622.0290	622.0290	414995	0.000
922.0098	922.0098	234109	0.000
1221.9906	1221.9906	98669	0.000
1521.9715	1521.9715	17586	-0.000
1821.9523	1821.9523	2160	-0.000

Figure S7: HRMS (ESI) copy for compound **3**.

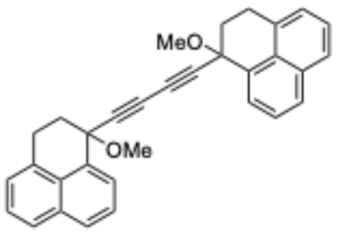

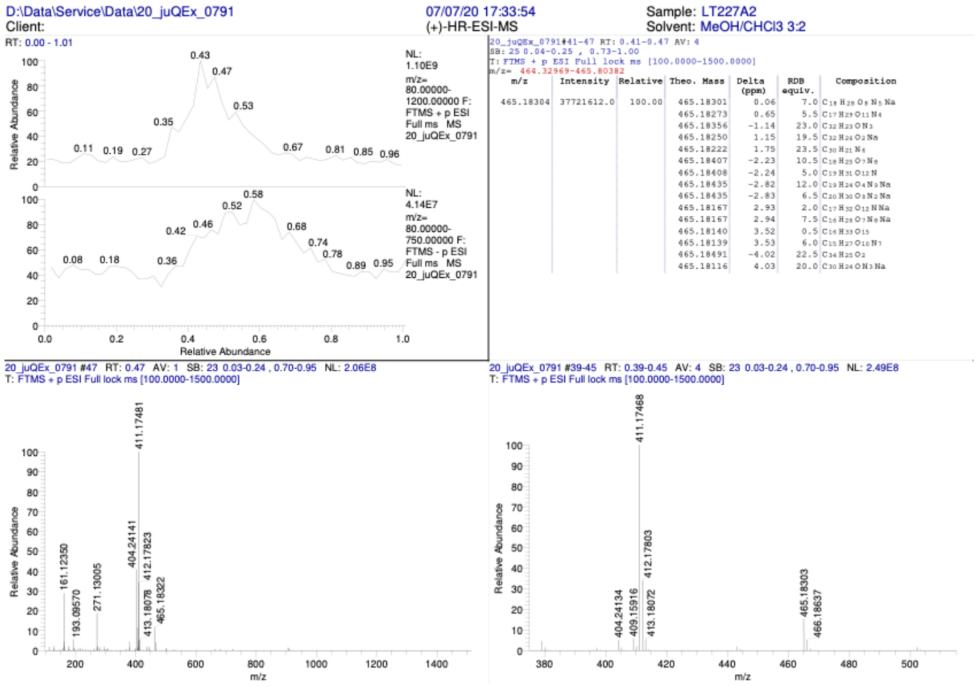

Figure S8: HRMS (ESI) copy for compound **4**.

II Methods: sample preparation and scanning probe experiments

STM and AFM measurements were conducted in a custom-built system operating under ultra-high vacuum (base pressure below 10^{-10} mbar) and at a temperature of 5 K. AFM measurements were performed with carbon monoxide (CO)-functionalized tips, and were performed in non-contact mode using a qPlus sensor[2] in frequency-modulation mode[3] with a 0.5 Å oscillation amplitude. STM measurements were performed with metallic or CO-functionalized tips. The STM data were acquired in both constant-current and constant-height modes, while the AFM data were acquired in constant-height mode with $V = 0$ V. Positive (negative) values of the tip height Δz indicate retraction (approach) from the STM set-point. The $dI/dV(V)$ spectra were obtained by numerical differentiation of the corresponding $I(V)$ spectra. STM and AFM images, and spectroscopy curves, were post-processed using Gaussian low-pass filters.

The Cu(111) surface was cleaned by repeated cycles of sputtering with Ne^+ ions and annealing to 800 K. NaCl was thermally evaporated on Cu(111) held at 283 K, which led to predominantly bilayer (100)-terminated islands, with a minority of third-layer islands (Fig. S9a). Submonolayer coverage of **1p** was achieved by flashing an oxidized silicon wafer with the molecules in front of the cold sample in the microscope (Fig. S9b). CO molecules for tip functionalization were dosed from the gas phase onto the cold surface.

To generate **1** from **1p**, the STM tip was positioned at the center of a phenalene unit at $V = 0.2$ V and $I = 1.0$ pA, and the feedback loop was opened. The tip was then retracted by ≥ 5 Å (to limit the tunneling current) and the voltage was increased to 4–5 V. This protocol resulted in the homolytic cleavage of the C(sp^3)–H bond at each phenalene unit, leading to the generation of **1** via the singly dehydrogenated intermediate **1′** (Fig. S11).

III Methods: theory

III.1 Ab-initio calculations

The geometry of **1** (Fig. S19) was optimized using unrestricted Kohn–Sham DFT at the PBE0/def2-TZVP level, as implemented in the ORCA 6.0.1 software package, without imposed symmetry constraints.[4,5] The optimized geometry revealed three distinct bond lengths in the central part of the molecule: 1.314 Å (bond b_A), 1.241 Å (bond b_B) and 1.358 Å (bond b_C). The central bond b_A is shorter than the terminal bond b_C, while the bond b_B is the shortest. To contextualize these bond lengths in terms of typical C−C single, double, and triple bonds at the PBE0 level of theory, we also optimized ethylene and propyne molecules. Ethylene exhibits a C−C bond length of 1.323 Å corresponding to a double bond, while propyne exhibits C−C bond lengths of 1.450 Å and 1.200 Å, corresponding to prototypical single and triple bonds, respectively.

To assess the impact of bond length on the diradical index (γ), we constructed three models in which the C−C bond lengths were fixed to that of standard C−C single, double, or triple bond values:

Model 1, in which the bond lengths of b_A–b_C were set equal to the C−C bond length of ethylene (cumulenic geometry),

Model 2, where the bond lengths of b_A and b_C were set to the bond length of the C−C single bond in propyne, while the bond length of b_B was set to that of the C−C triple bond in propyne (diynic geometry), and

Model 3, which retained the bond lengths obtained from the PBE0 geometry optimization (unconstrained geometry).

These models yield bond length alternation (BLA) (defined as $b_A - b_B$) of 0.000 Å, 0.250 Å, and 0.073 Å, respectively. The XYZ coordinates of Models 1–3 are provided in Tables S5–S7.

To assess the diradical character of **1**, we evaluated the singlet–triplet gap using different complete active space (CAS) sizes. The resulting energies were further corrected for dynamic correlation using domain-based local pair natural orbital N-electron valence perturbation theory to second order (DLPNO-NEVPT2).[6] A larger CAS is generally desirable, as a minimal CAS(2,2) includes only the frontier orbitals and therefore fails to capture all sources of exchange—particularly the Coulomb-driven exchange—leading to an incomplete description of the electronic structure.[7] The effect of active space size on the singlet-triplet gap is summarized in Table S1.

Table S1: Calculated SA-CASSCF and DLPNO-NEVPT2 singlet-triplet gaps of compound **1** (Model 3) with varying CAS. The SA-CASSCF calculation is averaged over two singlet and two triplet states.

**CAS**	**CASSCF** $S_0 \rightarrow T_1$ [eV]	**DLPNO-NEVPT2** $S_0 \rightarrow T_1$ [eV]
(2,2)	0.653	0.602
(4,4)	0.734	0.432
(6,6)	0.649	0.527
(8,8)	0.680	0.474
(10,10)	0.658	0.530

State-specific complete active space self-consistent field (CASSCF) calculations were performed in a CAS(12,12)[8] using the optimized geometries from all three models, thereby expanding the electronic wavefunction into all possible configuration state functions to determine the neutral singlet ground state. To accelerate the SCF procedure, the RIJCOSX approximation was employed, along with the def2/JK auxiliary basis set for exchange fitting.[9] Initial orbitals were generated from quasi-restricted natural orbitals obtained at the PBE0 level.

Following the definition of the minimal multiconfigurational representation of the wavefunction provided in the main manuscript (Eq. 1), we extracted the coefficients A_1 and A_2 corresponding to two spin determinants,[10] namely, the bonding ($A_1|\,222222000000\,\rangle$) and the antibonding ($A_2|\,222220200000\,\rangle$) configurations. In these kets, the sixth and seventh entries correspond to CASSCF natural orbitals that resemble the HOMO and LUMO (Fig. S20),

respectively, and each entry's value denotes the electron occupation of the corresponding orbital.

$$\text{Model 1 (cumulenic)} = -0.880432257\,|\,222222000000\,\rangle + 0.234255081\,|\,222220200000\,\rangle,$$

$$\text{Model 2 (diynic)} = -0.706808652\,|\,222222000000\,\rangle + 0.526172477\,|\,222220200000\,\rangle,$$

$$\text{Model 3 (unconstrained)} = -0.851882960\,|\,222222000000\,\rangle + 0.339288678\,|\,222220200000\,\rangle.$$

These coefficients were used to compute $\gamma = 2|\mathrm{A}_2|^2$ for each model (Table S2). Since our active space is CAS(12,12), rather than the minimal CAS(2,2) representation, we introduced a normalized quantity, γ_{norm}, which accounts for the total weight of both configurations. Specifically, we used the expression:

$$\gamma_{norm} = \frac{\gamma}{|\mathrm{A}_1|^2 + |\mathrm{A}_2|^2} \tag{S1}$$

The excited state energies for both the neutral and charged states were determined via state-averaged CASSCF (SA-CASSCF) calculations. The resulting energies were corrected for dynamic correlation using DLPNO-NEVPT2.[6] For the SA-CASSCF calculations, the neutral states were averaged over 8 states for both the triplet and singlet multiplicities. For the cationic and anionic states of the molecule, CAS(11,12) and CAS(13,12) were used, respectively, with 8 doublet excitations considered for each charge state. The selection of eight roots was based on the convergence instability we experienced when using two roots for Model 2 (positively and negatively charged states) and Model 3 (the positively charged states). In those cases, the NEVPT2 calculation presented false intruder states. To maintain consistency, we used eight roots for all calculations across the different excitation energies. Table S2 presents a summary of the results from these calculations.

Table S2: Summary of the CASSCF results for the diradical character γ and DLPNO-NEVPT2 excitation energies of the neutral and charged states.

**System**	γ	γ_{norm}	$S_0 \to S_1$ [eV]	$S_0 \to T_1$ [eV]	$D_0^+ \to D_1^+$ [eV]	$D_0^- \to D_1^-$ [eV]
Model 1 (cumul.)	0.11	0.13	1.637	0.734	1.103	1.171
Model 2 (diynic)	0.55	0.71	1.831	0.108	0.439	0.414
Model 3 (uncons.)	0.23	0.27	1.575	0.427	0.677	0.614

III.2 Model Hamiltonian and DMRG calculations

TB model and validation by DFT. To model **1**, we started by defining an effective tight-binding (TB) Hamiltonian. Although the C_4 chain features *sp* hybridization, we considered only the p_z orbital of each of the sp-hybridized carbon atoms. The reasoning behind this approximation is that, in a planar configuration, the p_z orbitals of the phenalenyl units only couple to the p_z orbitals of the chain, being orthogonal to the in-plane orbitals of the chain. DFT calculations confirmed that the remaining four electrons in the four in-plane orbitals of the chain hybridize and form orbitals far away ($\gtrsim$ 2 eV) from the Fermi energy. We thus disregard these in-plane orbitals in our model. As for the hoppings, we considered only nearest-neighbor terms, as the phenalenyl units are connected via their majority sublattice sites, where their unpaired electron wavefunction resides. In the phenalenyl units, we took the standard value for nanographenes, $t_1 = -2.7$ eV. In the carbon chain, we parameterized the hoppings according to the C-C distances, which we obtained from the three geometry optimizations described in the section above. In particular, we used the parameterization from Ref. 11, obtaining: (i) for the cumulenic case (where all bonds in the chain have C-C bond lengths equal to those of the double bonds obtained from ethylene), $t_{\mathrm{chain},b_\mathrm{A}} = t_{\mathrm{chain},b_\mathrm{B}} = t_{\mathrm{chain},b_\mathrm{C}} = -3.0$ eV; (ii) for the diynic case (where we assumed alternating single and triple

bonds, with C-C bond lengths obtained from propyne), $t_{\text{chain},b_\text{A}} = t_{\text{chain},b_\text{C}} = –2.6$ eV, $t_{\text{chain},b_\text{B}} = –3.5$ eV; (iii) for the unconstrained geometry optimization of the system, $t_{\text{chain},b_\text{A}} = –3.1$ eV, $t_{\text{chain},b_\text{B}} = –3.3$ eV, $t_{\text{chain},b_\text{C}} = –2.9$ eV. This parameterization was validated by comparing the TB energy levels and orbitals with DFT calculations, which yielded good agreement close to the Fermi energy (Fig. S13). DFT calculations were carried out with Quantum Espresso,[12] using the Perdew-Burke-Ernzerhof functional.[13] We used the pseudopotentials recommended for precision in the SSSP library[14] (version 1.3.0), together with the suggested kinetic energy cutoffs of 80 Ry for the wavefunction and 360 Ry for the charge density. To simulate the molecule, we considered a 14 Å vacuum separation between unit cells in all directions. For that reason, our grid of reciprocal points included only the Γ-point. We performed spin-restricted self-consistent field calculations until energies were converged up to 8×10^{-5} Ry, starting from the three optimized geometries described in section III.1.

Modeling interactions and DMRG details. Previous works on phenalenyl-based magnetic nanostructures[7,11,15] have shown that modeling interactions in this type of systems is difficult. On the one hand, methods based on the CAS approximation have convergence issues. For this reason, here we relied on density matrix renormalization group (DMRG)[16] calculations. On the other hand, the parameterization of the interaction parameters, especially their long-range behavior and dependence on the underlying surface, remains largely unknown. In light of this, here we adopted a simplified approach and modeled interactions via a Hubbard term with on-site Hubbard repulsion $U = 2|t_1|$. The resulting TB-Hubbard Hamiltonian was solved using DMRG as implemented in the ITensor library.[17] We performed DMRG sweeps with an adaptive scheme where the maximum bond dimensions were allowed to grow indefinitely in order to maintain the truncation error below 10^{-6}, which ensures high accuracy.

Using DMRG for the system with unconstrained geometry, we obtained a singlet-triplet ($S_0 \rightarrow T_1$) excitation energy of 361 meV, in reasonable agreement with the CASSCF calculations including perturbative corrections for dynamic correlations. Moreover, in the -1

charged sector, we obtained a doublet-doublet ($D_0^- \rightarrow D_1^-$) excitation energy of 568 meV, in good agreement not only with the DLPNO-NEVPT2-corrected CASSCF calculations but also with the experimental measurements.

Natural orbital analysis. Given a many-body state $|\psi\rangle$ obtained from DMRG, its natural orbitals and the corresponding occupation numbers were computed by diagonalizing the one-electron reduced density matrix,

$$\rho_{i,i'}^{(1)} = \langle\psi| \sum_{\sigma} \hat{c}_{i,\sigma}^{\dagger} \hat{c}_{i',\sigma} |\psi\rangle, \tag{S2}$$

where $\hat{c}_{i,\sigma}$ ($\hat{c}_{i,\sigma}^{\dagger}$) denotes the annihilation (creation) operators for an electron in site i with spin $\sigma = \uparrow, \downarrow$. In Fig. S21, we show the natural orbital analysis obtained for the ground state of the system with unconstrained geometry, found to be a singlet. These results corroborate the picture described in Eq. 1 of the main text, as only two natural orbitals have occupancies significantly different from 0 and 2, and their shapes resemble those of the HOMO and LUMO.

As an estimate of the number of unpaired electrons, we employed the expression

$$N_u = \sum_{\lambda} n_{\lambda}^2 (2 - n_{\lambda})^2, \tag{S3}$$

as outlined in Ref. 18, where n_{λ} are the occupation numbers of the natural orbitals. The ground-state results presented in Table S3 reveal the expected trend of increasing radical character from the cumulenic to the diynic geometry.

To obtain the coefficients A_1 and A_2 defined in Eq. 1 in the main text, we followed Ref. 11 and computed the overlap between the ground state obtained by DMRG and many-body states created by starting with a vacuum state and applying creation operators to fill the targeted natural orbitals. Results obtained for the three geometries are summarized in Table S3. Analogous overlap calculations were carried out to validate the scheme shown in

Table S3: Number of unpaired electrons N_u, multiconfigurational coefficients A_1 and A_2 defined in Eq. (1) of the main text, and diradical indices γ and γ_{norm}, all computed by DMRG for the ground state of the system with three different geometries.

**System**	N_u	A_1	A_2	$\gamma = 2\|A_2\|^2$	γ_{norm}
Model 1 (cumulenic)	0.60	−0.79	0.19	0.07	0.11
Model 2 (diynic)	1.26	−0.73	0.35	0.25	0.38
Model 3 (unconstrained)	0.71	0.78	−0.24	0.11	0.17

Fig. 3b of the main text.

Dyson orbitals. Dyson orbitals characterize transitions between states whose charge differs by ±1. They are particularly relevant to describe elastic excitations probed by scanning tunneling microscopy/spectroscopy (STM/STS) in multiconfigurational systems.[19] In this context, and disregarding population effects (e.g., due to temperature or bias), let us consider transitions between a neutral ground state with, say, N electrons, denoted by $|\psi_0^N\rangle$, to the m-th charged state with $N \pm 1$ electrons, denoted by $|\psi_m^{N\pm1}\rangle$. Such an excitation from $|\psi_0^N\rangle$ to $|\psi_m^{N\pm1}\rangle$ occurs at positive/negative bias, and corresponds to a negative/positive ion resonance (NIR/PIR), which we label as NIR+m/PIR−m. The corresponding spin-resolved ($\sigma = \uparrow, \downarrow$) Dyson orbitals are written as $\mathcal{D}^{\sigma}_{\mathrm{NIR}+m}(\boldsymbol{r})$ and $\mathcal{D}^{\sigma}_{\mathrm{PIR}-m}(\boldsymbol{r})$, where $\boldsymbol{r} = (x, y, z)$ denotes the spatial coordinates.

Since our model Hamiltonian preserves spin rotational invariance, the local density of the Dyson orbitals is independent of the spin, i.e., $|\mathcal{D}^{\sigma=\uparrow}_{\mathrm{NIR}+m/\mathrm{PIR}-m}(\boldsymbol{r})|^2 = |\mathcal{D}^{\sigma=\downarrow}_{\mathrm{NIR}+m/\mathrm{PIR}-m}(\boldsymbol{r})|^2$. Within our model framework, based on a carbon $2p_z$ atomic orbital basis and solved via DMRG, the Dyson orbitals can be expressed as

$$\mathcal{D}^{\sigma}_{\mathrm{NIR}+m}(\boldsymbol{r}) = \sum_i \mathcal{D}^{\sigma}_{\mathrm{NIR}+m}(i)\phi_i(\boldsymbol{r}), \tag{S4}$$

$$\mathcal{D}^{\sigma}_{\mathrm{PIR}-m}(\boldsymbol{r}) = \sum_i \mathcal{D}^{\sigma}_{\mathrm{PIR}-m}(i)\phi_i(\boldsymbol{r}), \tag{S5}$$

where

$$\phi_i(\boldsymbol{r}) = (z - z_i)\mathrm{e}^{-|\boldsymbol{r}-\boldsymbol{r}_i|/r_0}, \tag{S6}$$

with $r_0 = 0.325$ Å, denotes the $2p_z$ orbital of a carbon atom at site i, with position $\boldsymbol{r}_i = (x_i, y_i, z_i)$, and

$$\mathcal{D}^{\sigma}_{\mathrm{NIR}+m}(i) = \langle \psi^{N+1}_m | \hat{c}^{\dagger}_{\sigma}(i) | \psi^{N}_0 \rangle, \tag{S7}$$

$$\mathcal{D}^{\sigma}_{\mathrm{PIR}-m}(i) = \langle \psi^{N-1}_m | \hat{c}_{\sigma}(i) | \psi^{N}_0 \rangle. \tag{S8}$$

When accounting for population effects, for example, through a rate equation formalism (as described below), it may also be necessary to compute Dyson orbitals from excited states of the neutral system, for which the calculations are straightforward. In Fig. S22, we show the relevant Dyson orbitals for our system, computed using the many-body states with maximal S_z, where S_z denotes the total spin projection along the z axis. The corresponding local densities, with Clebsch-Gordan coefficients included (see section below), are shown in Fig. S23.

Rate equations. To reproduce the experimental results, we employed a rate-equation model using a steady-state master equation

$$0 = \sum_{i \neq j} \left(N_i \Gamma_{i \to j} - N_j \Gamma_{j \to i} \right) \tag{S9}$$

with $\Gamma_{i \to j}$ being the transition rate from state i to state j and N_i being the occupation probability of state i. We consider six states, as depicted in Fig. 3b in the main text and listed in Table S4. Their energies were chosen such that the calculated $\mathrm{d}I/\mathrm{d}V(V)$ spectrum (Fig. S24) matches the experimental one.

For the transition rates between these states we assume three pathways: charge transfer between molecule and surface Γ^S, charge transfer between tip and molecule Γ^T, and a charge-neutral decay rate from T_1 state to S_0. The coupling to the surface is modeled by a single

Table S4: Electronic states considered in the master equation.

**State**	**Energy** [eV]	**Charge**
S_0	0.00	0
T_1	0.30	0
D_0^-	0.60	$+1e^-$
D_1^-	1.30	$+1e^-$
D_0^+	1.65	$-1e^-$
D_1^+	2.50	$-1e^-$

tunneling barrier with height Φ_{eff} and width $d = 4$ Å, whereas the tunnel rate at $d = 0$ is defined to correspond to a quantum of conductance $2e/h$. The exact values for Γ^S are not crucial for the simulation, since the total current is limited by the coupling to the tip, i.e., $\Gamma^T \ll \Gamma^S$.

The coupling to the tip is based on the Dyson orbitals obtained by DMRG (see above) and the assumption of an s-wave tip. The low tunneling currents in experiment – and consequently large tip-sample distances – results in a predominant s-wave tunneling, even with a CO-functionalized tip.[20] To include the effect of a bias modulated tunneling barrier Φ_{eff}, the Dyson orbitals were calculated with Slater-type p_z orbitals at a constant-height plane 1.5 Å above the molecule and exponentially extrapolated into the vacuum. The effective tunneling barrier height is defined as $\Phi_{\text{eff}} = \Phi + \Delta E_{fi}\Delta Q_{fi} + eV/2$, with workfunction $\Phi = 4.9$ eV, bias voltage V and ΔE_{fi} and ΔQ_{fi} being the difference in energy and charge, respectively, between final and initial state. An empirical conversion factor η is used to translate the square of the extrapolated wavefunction into a hopping rate, that matches the experiment.

The effect of high electron-phonon coupling in the underlying NaCl surface[21–23] was captured by assuming a shift of $E_r = 0.2$ eV to higher energies for all charging transitions ($\Delta Q_{fi} \neq 0$) and a gaussian broadening with half width at half maximum of 0.12 eV.

To account for the different multiplicities of the states, all transition rates were multiplied with an additional factor Υ_{fi}, based on the Clebsch-Gordan coefficients. The transition from

singlet to doublet being $\Upsilon_{DS} = 2$, from doublet to triplet $\Upsilon_{TD} = 1.5$ and all other allowed transitions being $\Upsilon_{fi} = 1$. The master equation was solved for each respective tip position and voltage to obtain the steady-state occupation probabilities, and the tunneling current calculated from the net charge transfer between the molecule and surface. A good match with experiment was obtained for an empirical conversion factor of $\eta = 6 \times 10^9$ and a triplet lifetime of 30 ns. Note that the triplet lifetime in the simulations is based on several assumptions, e.g., s-wave tip or the precise relative intensities of orbitals at a given tip height. However, our experiments do not provide a direct measurement of intrinsic triplet lifetimes, which would be accessible by other methods using thicker insulating films.[24] The 30 ns used in our simulations can only be regarded as a rough estimate for the order of magnitude. Figure S24 shows representative calculated $I(V)$ and $\mathrm{d}I/\mathrm{d}V(V)$ spectra for a tip placed at $z = 11$ Å near the center of the molecule, comparable to the experimental spectrum shown in Fig. 3a of the main text. The calculated transition probability maps are displayed in Fig. S18 for $V = -1.8$ V, $z = 10.0$ Å; $V = +0.9$ V, $z = 10.3$ Å; and $V = +1.5$ V, $z = 11.5$ Å.

IV Experimental and theoretical data

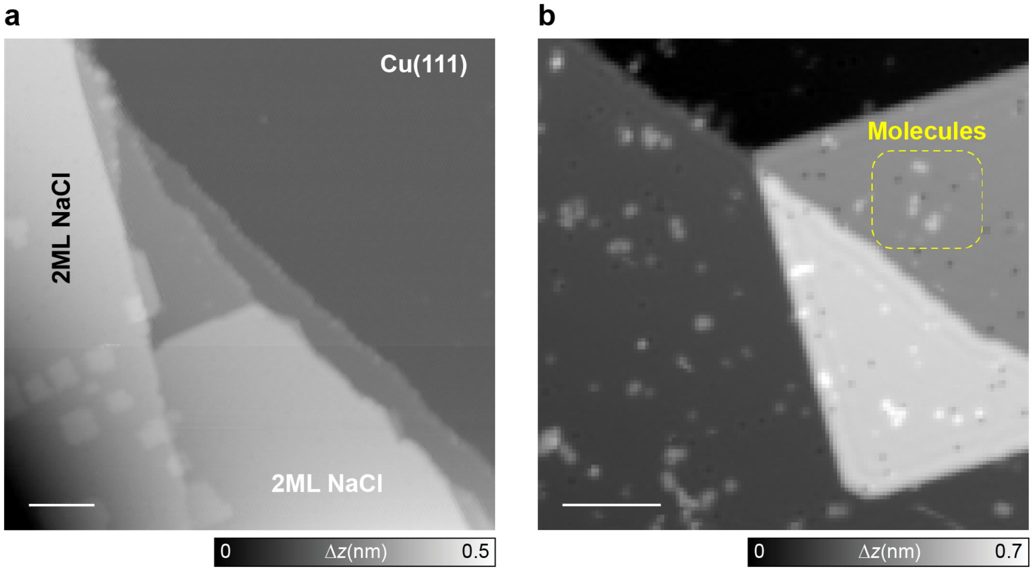

Figure S9: Overview STM images. (a) STM image of the NaCl/Cu(111) sample, showing predominantly bilayer (denoted 2ML) NaCl islands along with few third-layer islands. (b) STM image showing a submonalyer coverage of **1p** on NaCl/Cu(111). The yellow dashed rectangle highlights three **1p** molecules adsorbed on a bilayer NaCl island. CO molecules are imaged as small circular depressions on NaCl and Cu(111) surfaces. STM set-points: V = –0.7 V and I = 4.7 pA (a); V = 0.2 V and I = 2.8 pA (b). The data were acquired with a metallic tip. Scale bars: 10 nm.

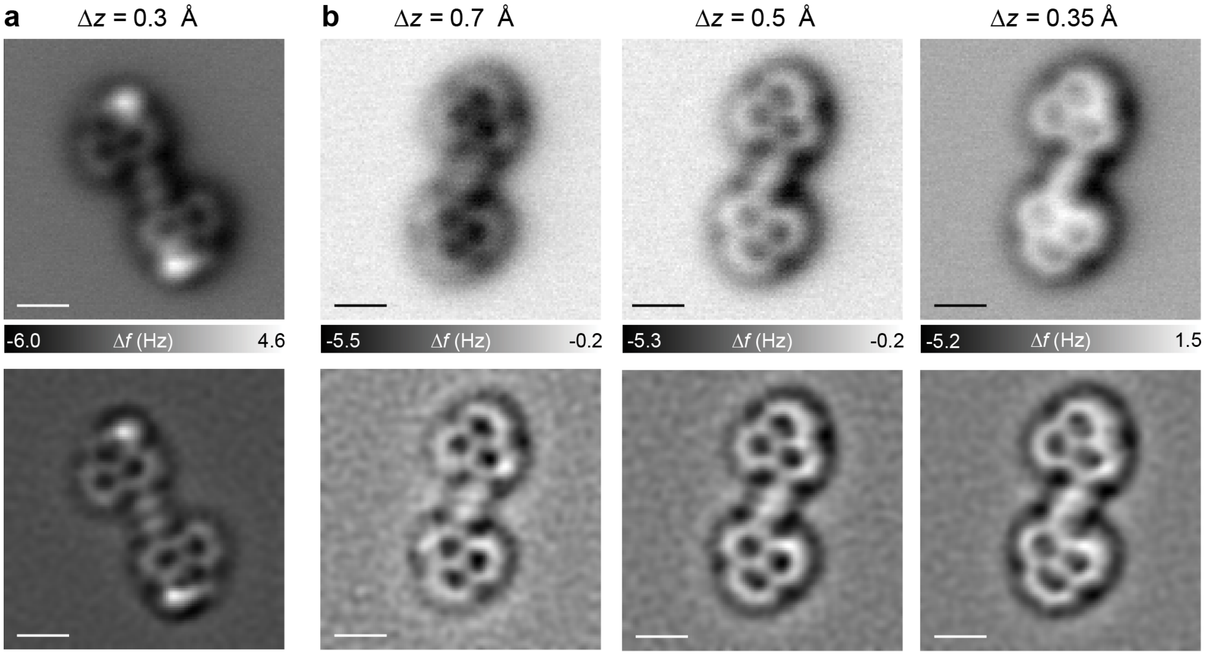

Figure S10: AFM images of (E)-**1p** (a) and (E)-**1** (b). Top: AFM images; bottom: corresponding Laplace-filtered versions. Qualitatively, the bond-order contrast in the C_4 chain of the E isomer appears similar to that of the Z isomer. STM set-point: $V = 0.2$ V and $I = 1.0$ pA on NaCl. Scale bars: 0.5 nm.

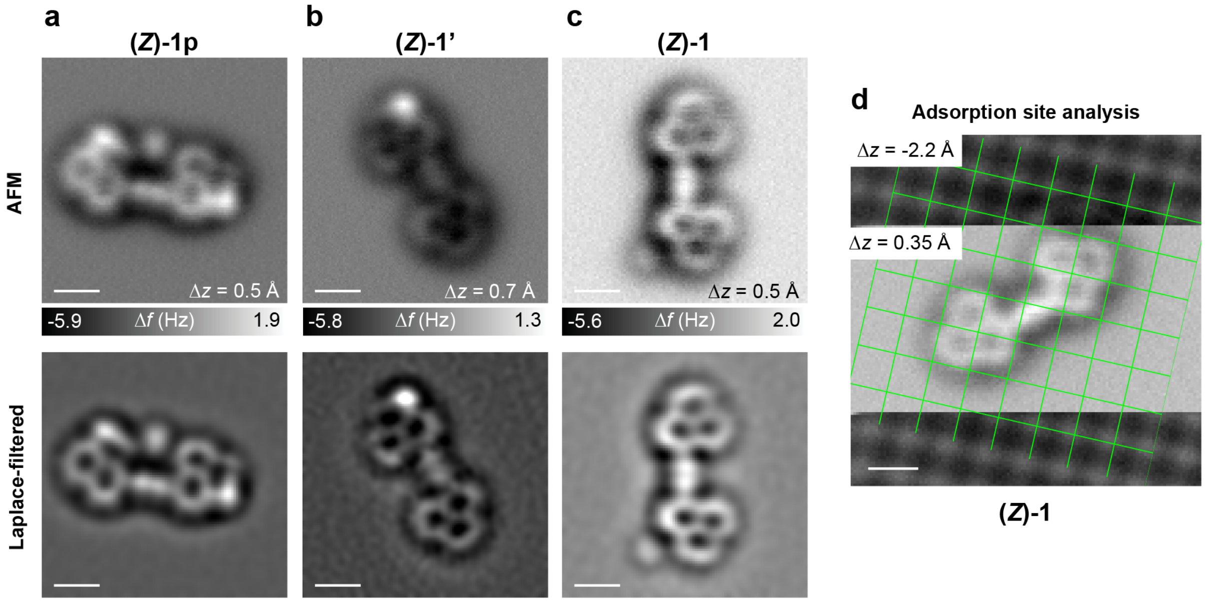

Figure S11: Stepwise dehydrogenation of (Z)-**1p**. (a–c) AFM images (top) with the corresponding Laplace-filtered images (bottom) of the precursor **1p** (a), the singly dehydrogenated intermediate **1′** (b) and the target molecule **1** (c). (d) AFM image where both the molecular structure of **1** and the NaCl lattice is resolved. Crossing points of the overlaid lattice (in green) correspond to the Cl^{-} sites of NaCl. STM set-point: $V = 0.2$ V and $I = 1.0$ pA on NaCl. Scale bars: 0.5 nm.

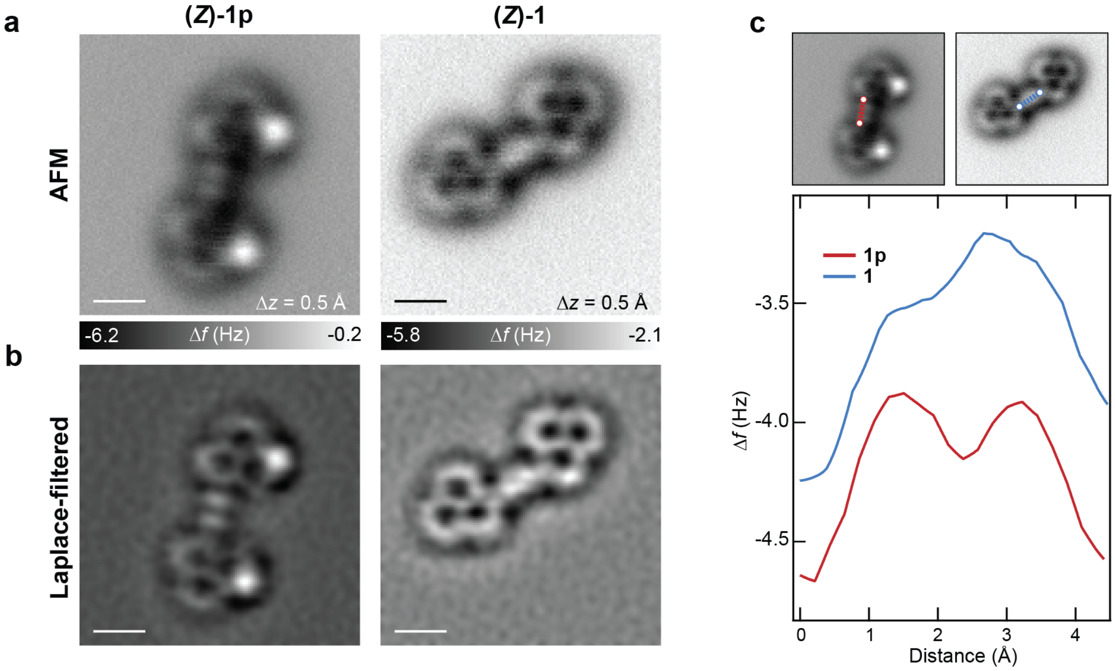

Figure S12: Bond-order contrast comparison with the same tip. (a, b) AFM images (top) and corresponding Laplace-filtered images (bottom) of (Z)-**1p** (a) and (Z)-**1** (b). (c) Δf line profiles along the C_4 chains of **1p** and **1**. STM set-point: $V = 0.2$ V and $I = 1.0$ pA on NaCl. Scale bars: 0.5 nm.

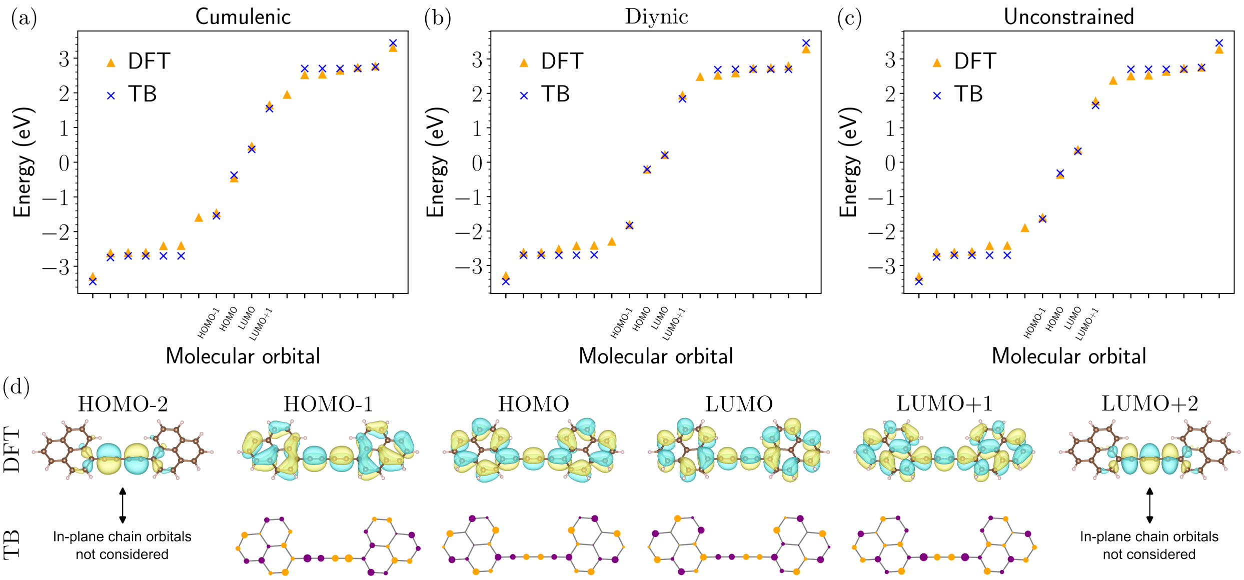

Figure S13: Validation of TB model by DFT. (a–c) DFT and TB energy levels, obtained for (a) cumulenic, (b) diynic, and (c) unconstrained geometries. States above and below zero energy are unoccupied and occupied, respectively. TB energy levels were shifted (laterally) at the HOMO-2 and LUMO+2 positions, as the corresponding DFT orbitals were found to be predominantly composed of in-plane orbitals of the C_4 sp-hybridized chain, not considered at the TB level. (d) DFT and TB molecular orbitals for the system with unconstrained geometry.

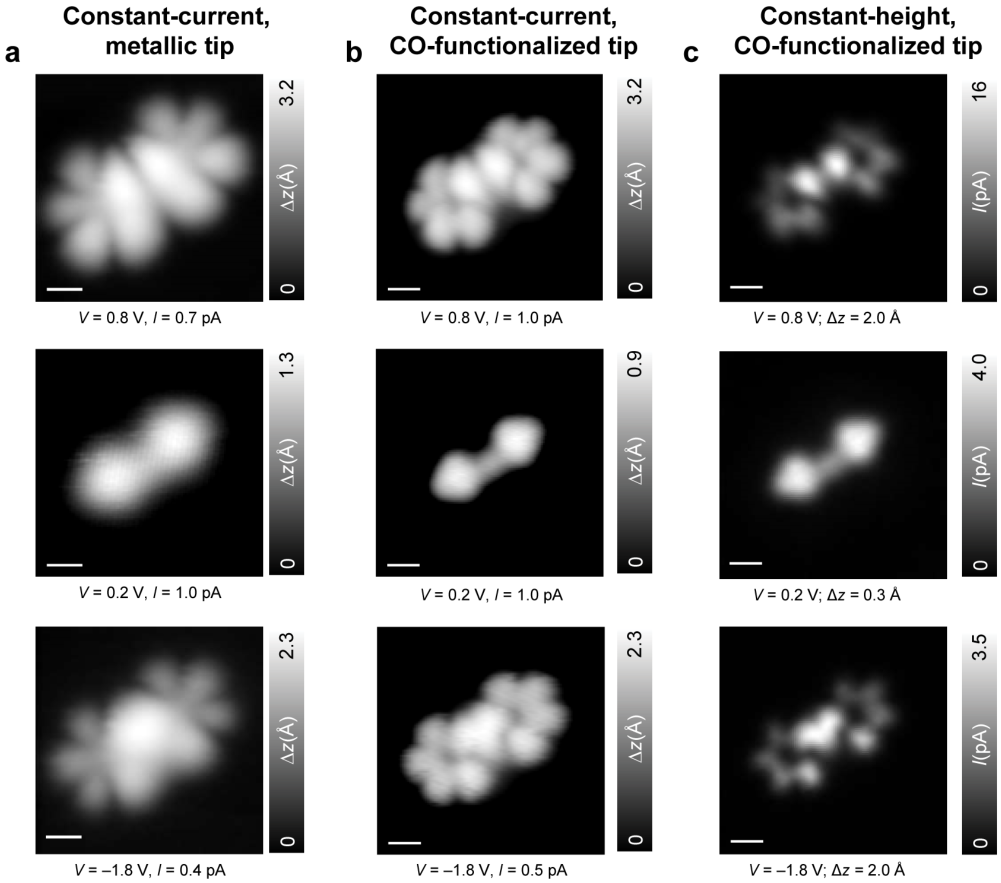

Figure S14: STM images at the negative ion resonance (top), in gap (middle) and positive ion resonance (bottom) of (Z)-**1**, acquired with metallic (a) and CO-functionalized (b, c) tips. The set-point current for constant-height STM images was $I = 0.5$ pA on NaCl, while the set-point voltages were the same as indicated for the respective panels. Scale bars: 0.5 nm.

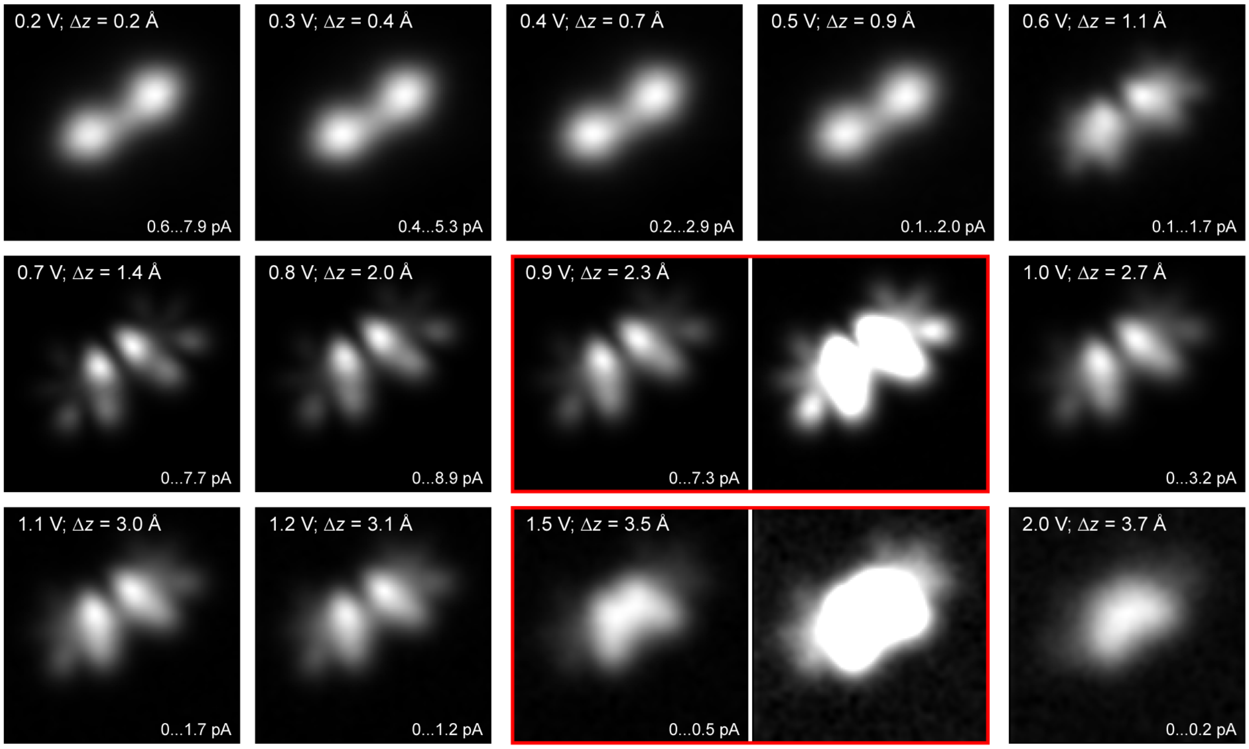

Figure S15: Voltage-dependent STM images of (Z)-**1** at positive bias. STM images at the first two negative ion resonances (V = 0.9 and 1.5 V) are highlighted in red. To resolve LDOS features at both the C_4 chain and the phenalenyl units, images at V = 0.9 and 1.5 V are shown with two contrast levels. All images were acquired with a metallic tip. The current setpoint was I = 0.5 pA on NaCl, while the set-point voltages were the same as indicated for the respective panels. The minimum and maximum values of the current are indicated for each panel. Image sizes: 3.2 nm × 3.2 nm.

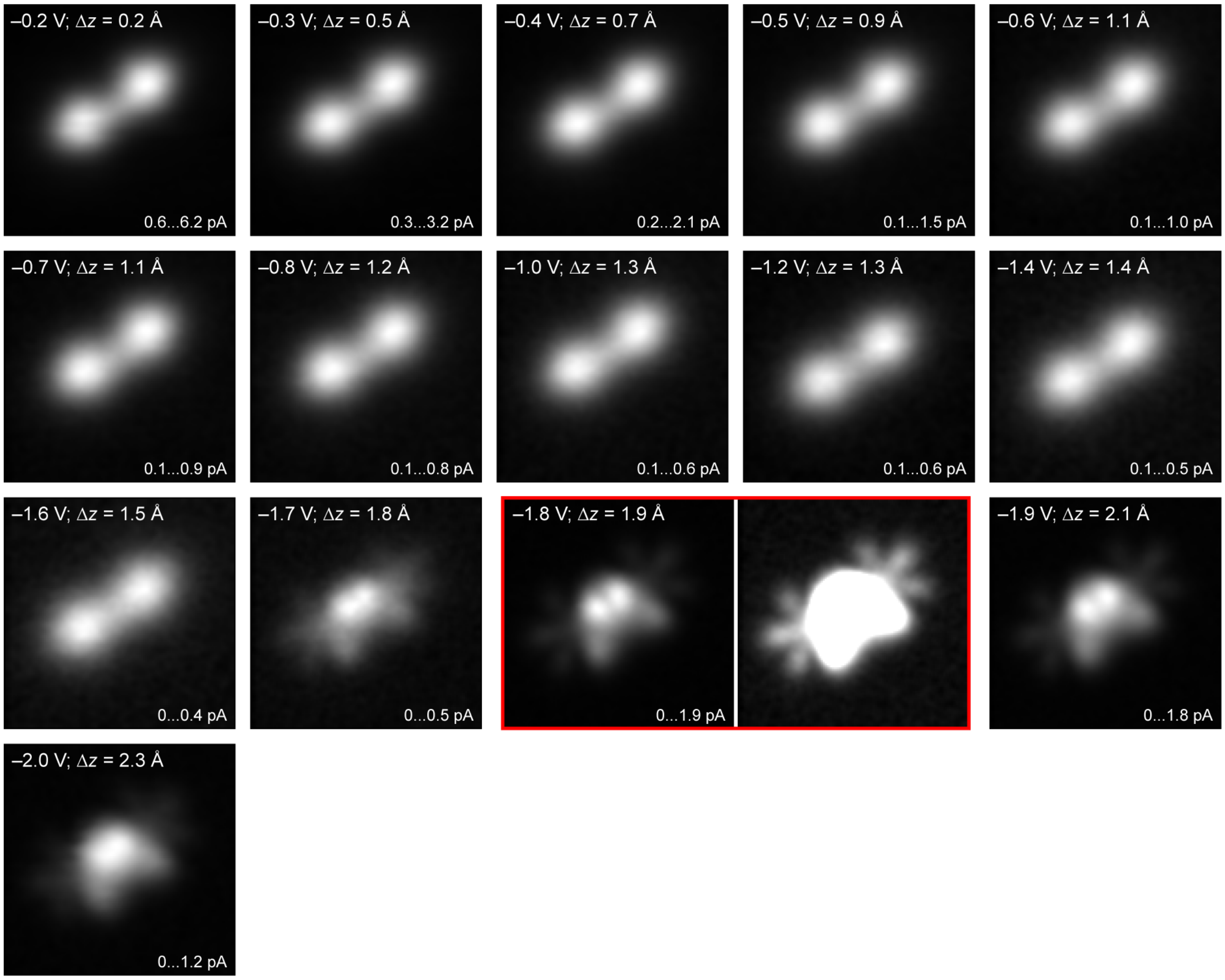

Figure S16: Voltage-dependent STM images of (Z)-**1** at negative bias. STM image at the positive ion resonance (V = –1.8 V) is highlighted in red. To resolve LDOS features at both the C_4 chain and the phenalenyl units, the image at V = –1.8 V is shown with two contrast levels. All images were acquired with a metallic tip. The set-point current was I = 1.0 pA on NaCl, while the set-point voltages were the same as indicated for the respective panels. The minimum and maximum values of the current are indicated for each panel. Image sizes: 3.2 nm × 3.2 nm

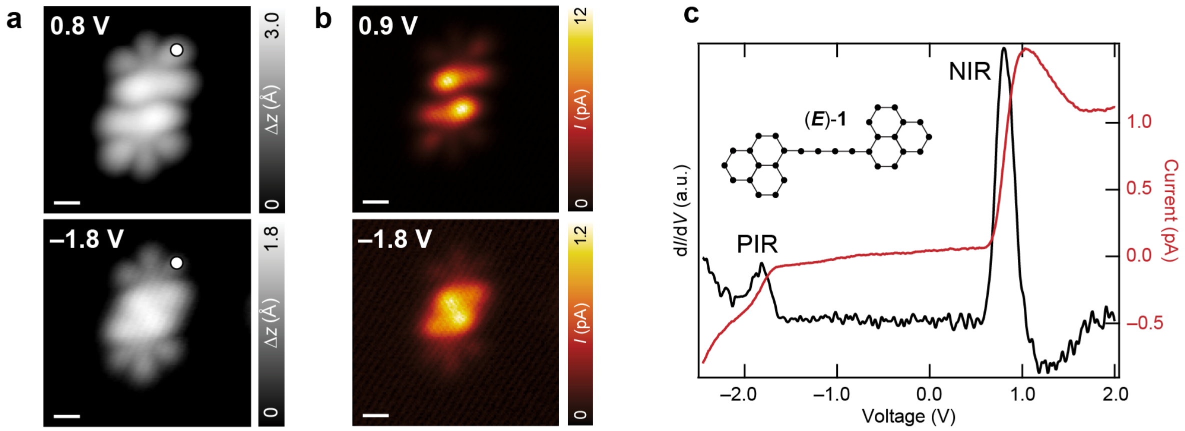

Figure S17: Electronic characterization of (E)-**1**. (a, b) Constant-current (a) and constant-height (b) STM images of (E)-**1** at the negative (top) and positive (bottom) ion resonances. Although the STM images of (E)-**1** appear different from that of the Z isomer because of the different molecular geometry, they show the same essential features as observed for the Z isomer, namely, a dominant nodal plane at the center of the C_4 chain (0.8 V), and maximum at the center of the C_4 chain along with a weak depression reminiscent of a nodal plane (–1.8 V). The set-point current was $I = 1.0$ pA, with the feedback opened on NaCl for the images in (b). The set-point voltages were the same as indicated for the respective panels. For the images in (b), $\Delta z = 2.0$ Å (0.9 V) and 1.8 Å (–1.8 V). (c) $I(V)$ spectrum and the corresponding $dI/dV(V)$ spectrum acquired on **1** at the position indicated by the filled white circles in (a) (open feedback parameters: $V = $ –2.5 V, $I = 1.0$ pA). The energetic positions of the positive and negative ion resonances of (E)-**1** are identical to the Z isomer. All images and spectroscopy data were acquired with a metallic tip. Scale bars: 0.5 nm.

Failure of the single-particle description. The constant-height STM images at the PIR and NIR of **1**, shown in Fig. 3 and Fig. S18a, exhibit a dominant bonding–antibonding character that could be interpreted as the HOMO and LUMO densities of the closed-shell molecule. Using a simple TB approach, the calculated HOMO and LUMO orbitals (Fig. S18b) somewhat agree with the experimentally observed features, apart from the weak depression reminiscent of a nodal plane in the STM image at the PIR, which is attributed to transition involving the neutral triplet state. In addition, a notable discrepancy arises for the NIR+1 measured at 1.5 V: the experimental STM image at the NIR+1 in Fig. S18a does not match either the calculated LUMO+1 LDOS map, or the LDOS map corresponding to a superposition of the LUMO and LUMO+1 in Fig. S18b.

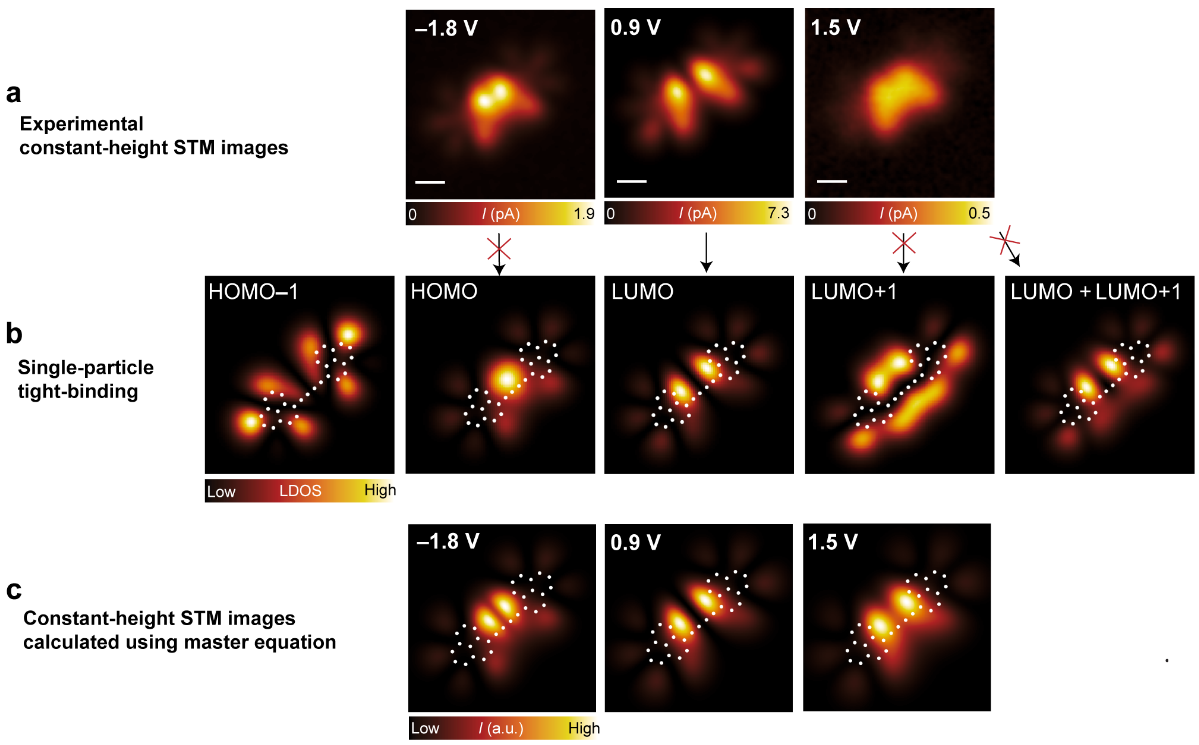

Figure S18: Assignment of STM images at the ion resonances to orbital densities. (a) Experimental constant-height STM images at the ion resonances (reported in the main text). (b) Calculated LDOS maps of the frontier molecular orbitals using TB level of theory ($U = 0$ eV). (c) Calculated transition probability maps using master equation at the experimental voltages. Scale bars: 0.5 nm (applies also to the images in panels b and c).

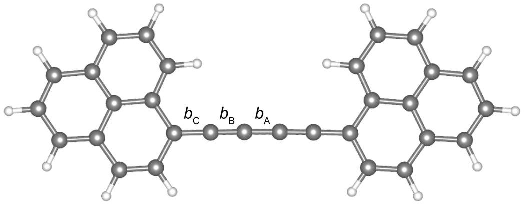

Figure S19: Molecular structure of **1**. The three bonds b_A–b_C in the central part of the molecule are labeled in accordance with Fig. 1 in the main text.

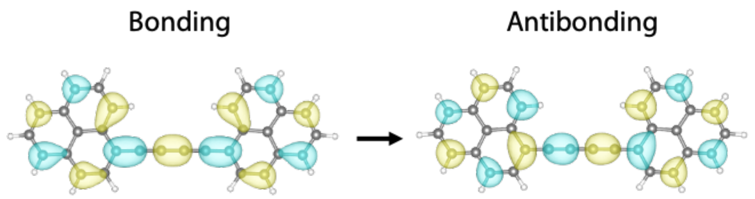

Figure S20: The natural orbitals obtained from SA-CASSCF calculations provide a detailed description of the electronic excitations in both the neutral and charged states. In these states, the primary excitations involve the orbitals with bonding and antibonding symmetries, consistent with the schematic presented in the main text (Fig. 3b).

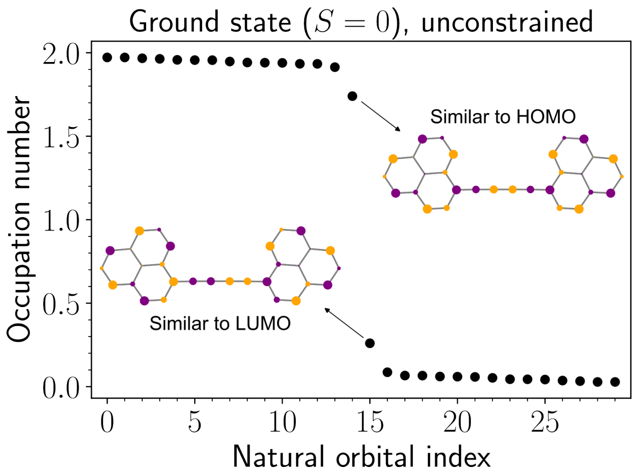

Figure S21: Natural orbital analysis for the ground state of the system with unconstrained geometry. Occupation numbers of natural orbitals are computed by DMRG. Insets show the natural orbitals with the most fractional occupancies (i.e., those with occupancies furthest from 0 and 2), whose shapes resemble the HOMO and LUMO (cf. Fig. S13d).

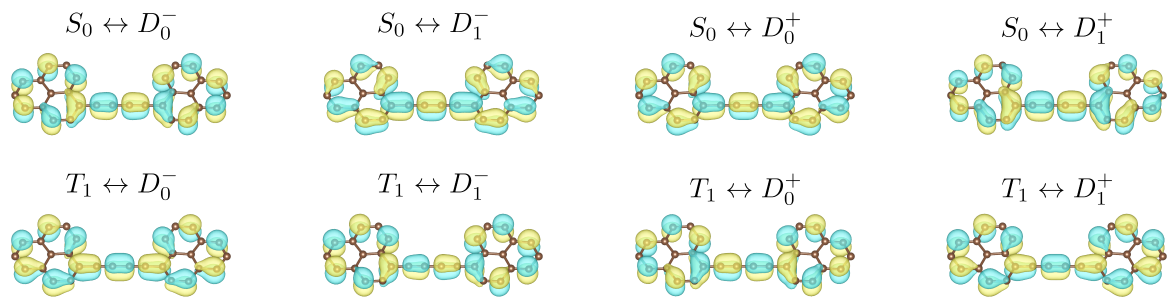

Figure S22: DMRG calculated Dyson orbitals of **1** (for the unconstrained geometry), corresponding to all transitions between the considered neutral and charged states.

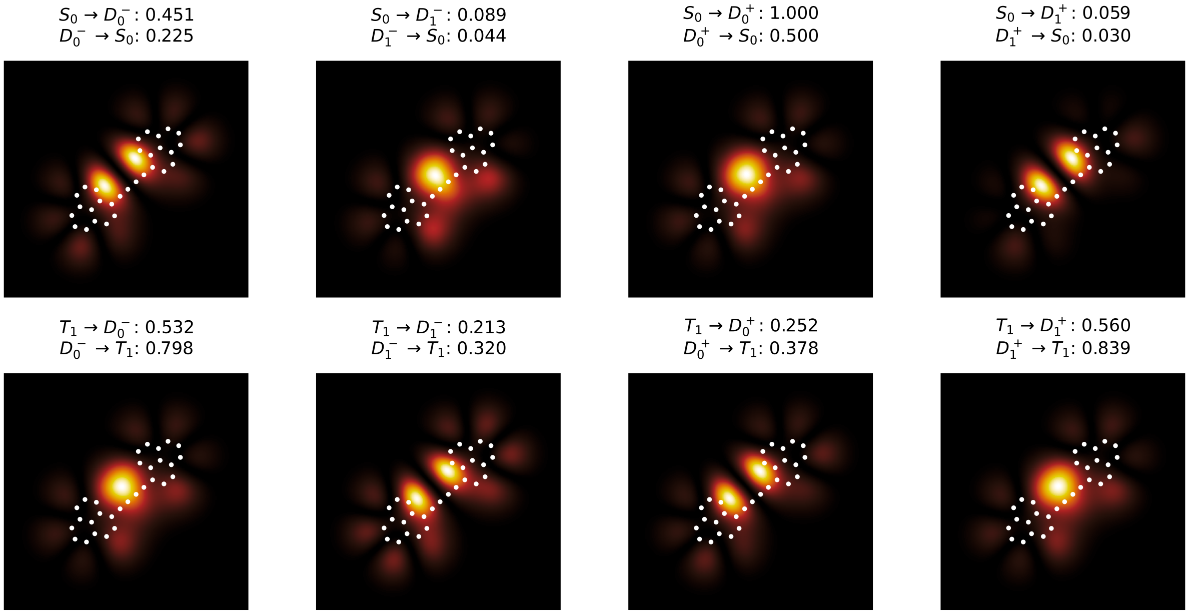

Figure S23: Constant-height LDOS maps of Dyson orbitals, corresponding to all transitions between the considered neutral and charged states. The maps were calculated by DMRG for the unconstrained geometry using Slater-type p_z orbitals at a height of $z = 20\,\text{Å}$ above the molecule. The relative intensities listed above the maps, which already account for the Clebsch-Gordan coefficients, are determined by the maximal intensity of each map, divided by the maximum of the $S_0 \rightarrow D_0^+$ transition. Image sizes: 2.9 nm × 2.8 nm.

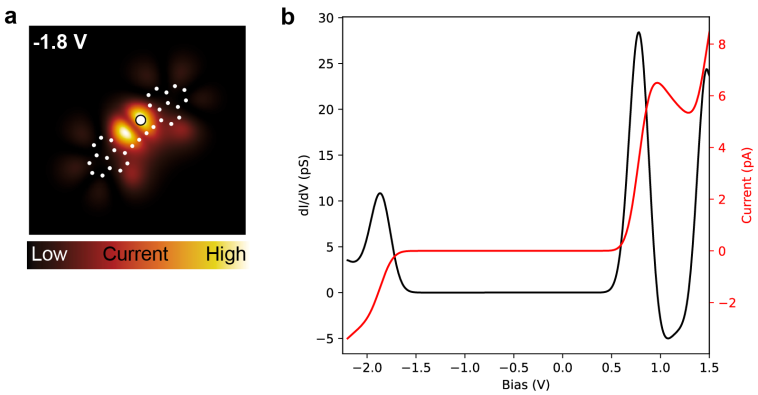

Figure S24: Calculated electronic structure of (Z)-**1** using the master equation. (a) Transition probability map at $-1.8\,\mathrm{V}$ and $z = 10\,\text{Å}$. (b) $I(V)$ spectrum and the corresponding $\mathrm{d}I/\mathrm{d}V(V)$ spectrum for the position indicated by the white filled circle in (a) and at a height of $z = 11\,\text{Å}$. Image size: 2.9 nm $\times$ 2.8 nm.

Table S5: XYZ coordinates of **1** (Model 1, cumulenic) (Å)

C	18.397286	15.383297	7.502757
C	19.805864	15.506288	7.502480
C	20.600655	14.329505	7.502806
C	19.965129	13.057964	7.503256
C	18.580640	12.987541	7.503525
C	17.801169	14.148460	7.503298
C	22.017320	14.434882	7.502566
C	22.597947	15.697016	7.501962
C	21.814334	16.849327	7.501616
C	20.442503	16.762699	7.501867
C	20.784508	11.881411	7.503270
C	22.134147	11.957695	7.503030
C	22.812898	13.217867	7.502828
C	24.135642	13.244390	7.502795
C	25.458644	13.248843	7.502794
C	26.781654	13.248430	7.502787
C	28.104653	13.242998	7.502753
C	29.427401	13.216662	7.502709
C	30.222651	14.433939	7.503003
C	31.639356	14.328960	7.502839
C	32.275254	13.057604	7.502377
C	31.456181	11.880831	7.502178
C	30.106511	11.956697	7.502443
C	32.433798	15.505973	7.503168
C	31.796823	16.762219	7.503701
C	30.424974	16.848471	7.503860
C	29.641685	15.695934	7.503495
C	33.659768	12.987613	7.502197
C	34.438903	14.148765	7.502507
C	33.842409	15.383417	7.503003
H	19.833407	17.660176	7.501575
H	22.295777	17.820502	7.501091
H	23.679005	15.777502	7.501727
H	22.738108	11.058545	7.503033
H	20.295046	10.913348	7.503485
H	28.560609	15.776143	7.503646
H	29.943259	17.819512	7.504269
H	32.405686	17.659867	7.503987
H	31.945920	10.912917	7.501841
H	29.502825	11.057368	7.502314
H	34.139315	12.014540	7.501820
H	35.519832	14.068383	7.502400
H	34.444212	16.285779	7.503280
H	18.101393	12.014317	7.503895
H	16.720270	14.067703	7.503527
H	17.795184	16.285460	7.502500

Table S6: XYZ coordinates of **1** (Model 2, diynic) (Å)

C	18.397286	15.383297	7.502757
C	19.805864	15.506288	7.502480
C	20.600655	14.329505	7.502806
C	19.965129	13.057964	7.503256
C	18.580640	12.987541	7.503525
C	17.801169	14.148460	7.503298
C	22.017320	14.434882	7.502566
C	22.597947	15.697016	7.501962
C	21.814334	16.849327	7.501616
C	20.442503	16.762699	7.501867
C	20.784508	11.881411	7.503270
C	22.134147	11.957695	7.503030
C	22.812898	13.217867	7.502828
C	24.262206	13.246928	7.502792
C	25.462199	13.250967	7.502791
C	26.911799	13.250514	7.502784
C	28.111789	13.245588	7.502753
C	29.561102	13.216732	7.502705
C	30.356352	14.434008	7.502999
C	31.773057	14.329030	7.502834
C	32.408955	13.057674	7.502372
C	31.589882	11.880900	7.502173
C	30.240212	11.956766	7.502439
C	32.567499	15.506042	7.503164
C	31.930524	16.762288	7.503697
C	30.558675	16.848540	7.503855
C	29.775387	15.696003	7.503490
C	33.793470	12.987682	7.502192
C	34.572604	14.148834	7.502503
C	33.976110	15.383487	7.502998
H	19.833407	17.660176	7.501575
H	22.295777	17.820502	7.501091
H	23.679005	15.777502	7.501727
H	22.738108	11.058545	7.503033
H	20.295046	10.913348	7.503485
H	28.694310	15.776212	7.503641
H	30.076960	17.819581	7.504265
H	32.539387	17.659936	7.503982
H	32.079621	10.912987	7.501837
H	29.636526	11.057438	7.502309
H	34.273016	12.014609	7.501816
H	35.653533	14.068452	7.502395
H	34.577913	16.285849	7.503275
H	18.101393	12.014317	7.503895
H	16.720270	14.067703	7.503527
H	17.795184	16.285460	7.502500

Table S7: XYZ coordinates of **1** (Model 3, unconstrained) (Å)

C	19.805864	15.506288	7.502480
C	20.442503	16.762699	7.501867
C	21.814334	16.849327	7.501616
C	22.597947	15.697016	7.501962
C	22.017320	14.434882	7.502566
C	22.812898	13.217867	7.502828
C	22.134148	11.957695	7.503030
C	20.784508	11.881411	7.503270
C	24.171126	13.245102	7.502794
C	25.412360	13.249280	7.502793
C	26.726561	13.248869	7.502787
C	27.967794	13.243774	7.502755
C	29.326028	13.216731	7.502710
C	30.121277	14.434008	7.503004
C	29.540312	15.696002	7.503495
C	30.323600	16.848539	7.503860
C	31.695450	16.762288	7.503701
C	32.332424	15.506042	7.503169
C	31.537982	14.329029	7.502839
C	32.173880	13.057674	7.502377
C	31.354807	11.880900	7.502178
C	30.005138	11.956766	7.502444
C	33.558395	12.987682	7.502197
C	34.337530	14.148834	7.502507
C	33.741036	15.383487	7.503003
C	20.600655	14.329505	7.502806
C	19.965129	13.057964	7.503256
C	18.580640	12.987541	7.503525
C	17.801170	14.148460	7.503298
C	18.397286	15.383297	7.502757
H	19.833407	17.660176	7.501575
H	22.295777	17.820502	7.501091
H	23.679005	15.777502	7.501727
H	22.738108	11.058545	7.503033
H	20.295046	10.913348	7.503485
H	28.459235	15.776211	7.503646
H	29.841885	17.819580	7.504269
H	32.304312	17.659936	7.503987
H	31.844547	10.912987	7.501842
H	29.401452	11.057437	7.502315
H	34.037942	12.014609	7.501821
H	35.418459	14.068452	7.502400
H	34.342838	16.285849	7.503280
H	18.101393	12.014317	7.503895
H	16.720270	14.067703	7.503527
H	17.795184	16.285460	7.502500

References

(1) Fulmer, G. R.; Miller, A. J. M.; Sherden, N. H.; Gottlieb, H. E.; Nudelman, A.; Stoltz, B. M.; Bercaw, J. E.; Goldberg, K. I. NMR Chemical Shifts of Trace Impurities: Common Laboratory Solvents, Organics, and Gases in Deuterated Solvents Relevant to the Organometallic Chemist. *Organometallics* **2010**, *29*, 2176–2179.

(2) Giessibl, F. J. High-speed force sensor for force microscopy and profilometry utilizing a quartz tuning fork. *Applied Physics Letters* **1998**, *73*, 3956–3958.

(3) Albrecht, T. R.; Grütter, P.; Horne, D.; Rugar, D. Frequency modulation detection using high-Q cantilevers for enhanced force microscope sensitivity. *Journal of Applied Physics* **1991**, *69*, 668–673.

(4) Neese, F. The ORCA program system. *WIREs Computational Molecular Science* **2012**, *2*, 73–78.

(5) Neese, F. Software update: The ORCA program system—Version 5.0. *WIREs Computational Molecular Science* **2022**, *12*, e1606.

(6) Guo, Y.; Sivalingam, K.; Valeev, E. F.; Neese, F. SparseMaps—A systematic infrastructure for reduced-scaling electronic structure methods. III. Linear-scaling multireference domain-based pair natural orbital N-electron valence perturbation theory. *The Journal of Chemical Physics* **2016**, *144*, 094111.

(7) Jacob, D.; Fernández-Rossier, J. Theory of intermolecular exchange in coupled spin- 1 2 nanographenes. *Physical Review B* **2022**, *106*, 205405.

(8) Kollmar, C.; Sivalingam, K.; Helmich-Paris, B.; Angeli, C.; Neese, F. A perturbation-based super-CI approach for the orbital optimization of a CASSCF wave function. *Journal of Computational Chemistry* **2019**, *40*, 1463–1470.

(9) Neese, F.; Wennmohs, F.; Hansen, A.; Becker, U. Efficient, approximate and parallel Hartree–Fock and hybrid DFT calculations. A 'chain-of-spheres' algorithm for the Hartree–Fock exchange. *Chemical Physics* **2009**, *356*, 98–109, Moving Frontiers in Quantum Chemistry:.

(10) Ugandi, M.; Roemelt, M. A recursive formulation of one-electron coupling coefficients for spin-adapted configuration interaction calculations featuring many unpaired electrons. *International Journal of Quantum Chemistry* **2023**, *123*, e27045.

(11) Catarina, G.; Turco, E.; Krane, N.; Bommert, M.; Ortega-Guerrero, A.; Gröning, O.; Ruffieux, P.; Fasel, R.; Pignedoli, C. A. Conformational Tuning of Magnetic Interactions in Coupled Nanographenes. *Nano Letters* **2024**, *24*, 12536–12544.

(12) Giannozzi, P. et al. QUANTUM ESPRESSO: a modular and open-source software project for quantum simulations of materials. *Journal of Physics: Condensed Matter* **2009**, *21*, 395502.

(13) Perdew, J. P.; Burke, K.; Ernzerhof, M. Generalized Gradient Approximation Made Simple. *Physical Review Letters* **1996**, *77*, 3865–3868.

(14) Prandini, G.; Marrazzo, A.; Castelli, I. E.; Mounet, N.; Marzari, N. Precision and efficiency in solid-state pseudopotential calculations. *npj Computational Materials* **2018**, *4*, 1–13.

(15) Krane, N.; Turco, E.; Bernhardt, A.; Jacob, D.; Gandus, G.; Passerone, D.; Luisier, M.; Juríček, M.; Fasel, R.; Fernández-Rossier, J.; Ruffieux, P. Exchange Interactions and Intermolecular Hybridization in a Spin-1/2 Nanographene Dimer. *Nano Letters* **2023**, *23*, 9353–9359.

(16) White, S. R. Density matrix formulation for quantum renormalization groups. *Physical Review Letters* **1992**, *69*, 2863–2866.

(17) Fishman, M.; White, S.; Stoudenmire, E. The ITensor Software Library for Tensor Network Calculations. *SciPost Physics Codebases* **2022**, 004.

(18) Head-Gordon, M. Characterizing unpaired electrons from the one-particle density matrix. *Chemical Physics Letters* **2003**, *372*, 508–511.

(19) Kumar, M.; Soler-Polo, D.; Lozano, M.; Monino, E.; Veis, L.; Jelinek, P. Multireference Theory of Scanning Tunneling Spectroscopy Beyond One-Electron Molecular Orbitals: Can We Image Molecular Orbitals? *Journal of the American Chemical Society* **2025**, *147*, 24993–25003.

(20) Paschke, F.; Lieske, L.-A.; Albrecht, F.; Chen, C. J.; Repp, J.; Gross, L. Distance and Voltage Dependence of Orbital Density Imaging Using a CO-Functionalized Tip in Scanning Tunneling Microscopy. *ACS Nano* **2025**, *19*, 2641–2650.

(21) Fatayer, S.; Schuler, B.; Steurer, W.; Scivetti, I.; Repp, J.; Gross, L.; Persson, M.; Meyer, G. Reorganization energy upon charging a single molecule on an insulator measured by atomic force microscopy. *Nature Nanotechnology* **2018**, *13*, 376–380.

(22) Vasilev, K.; Canola, S.; Scheurer, F.; Boeglin, A.; Lotthammer, F.; Chérioux, F.; Neuman, T.; Schull, G. Exploring the Role of Excited States' Degeneracy on Vibronic Coupling with Atomic-Scale Optics. *ACS Nano* **2024**, *18*, 28052–28059.

(23) Sellies, L.; Eckrich, J.; Gross, L.; Donarini, A.; Repp, J. Controlled single-electron transfer enables time-resolved excited-state spectroscopy of individual molecules. *Nature Nanotechnology* **2025**, *20*, 27–35.

(24) Peng, J.; Sokolov, S.; Hernangómez-Pérez, D.; Evers, F.; Gross, L.; Lupton, J. M.; Repp, J. Atomically resolved single-molecule triplet quenching. *Science* **2021**, *373*, 452–456.
